# Microbial Processing of Jellyfish Detritus in the Ocean

**DOI:** 10.3389/fmicb.2020.590995

**Published:** 2020-10-30

**Authors:** Tinkara Tinta, Zihao Zhao, Alvaro Escobar, Katja Klun, Barbara Bayer, Chie Amano, Luca Bamonti, Gerhard J. Herndl

**Affiliations:** ^1^Department of Functional and Evolutionary Ecology, Bio-Oceanography Unit, Faculty of Life Sciences, University of Vienna, Vienna, Austria; ^2^Marine Biology Station Piran, National Institute of Biology, Piran, Slovenia; ^3^Vienna Metabolomics Center, University of Vienna, Vienna, Austria; ^4^Department of Marine Microbiology and Biogeochemistry, Royal Netherlands Institute for Sea Research (NIOZ), Utrecht University, Den Burg, Netherlands

**Keywords:** jellyfish blooms, detritus, dissolved organic matter, marine microbial community, biodegradation, proteomics, biogeochemical cycles

## Abstract

When jellyfish blooms decay, sinking jellyfish detrital organic matter (jelly-OM), rich in proteins and characterized by a low C:N ratio, becomes a significant source of OM for marine microorganisms. Yet, the key players and the process of microbial jelly-OM degradation and the consequences for marine ecosystems remain unclear. We simulated the scenario potentially experienced by the coastal pelagic microbiome after the decay of a bloom of the cosmopolitan *Aurelia aurita* s.l. We show that about half of the jelly-OM is instantly available as dissolved organic matter and thus, exclusively and readily accessible to microbes. During a typical decay of an *A. aurita* bloom in the northern Adriatic Sea about 100 mg of jelly-OM L^–1^ becomes available, about 44 μmol L^–1^ as dissolved organic carbon (DOC), 13 μmol L^–1^ as total dissolved nitrogen, 11 μmol L^–1^ of total hydrolyzable dissolved amino acids (THDAA) and 0.6 μmol L^–1^ PO_4_^3–^. The labile jelly-OM was degraded within 1.5 days (>98% of proteins, ∼70% of THDAA, 97% of dissolved free amino acids and the entire jelly-DOC pool) by a consortium of *Pseudoalteromonas*, *Alteromonas*, and *Vibrio*. These bacteria accounted for >90% of all metabolically active jelly-OM degraders, exhibiting high bacterial growth efficiencies. This implies that a major fraction of the detrital jelly-OM is rapidly incorporated into biomass by opportunistic bacteria. Microbial processing of jelly-OM resulted in the accumulation of tryptophan, dissolved combined amino acids and inorganic nutrients, with possible implications for biogeochemical cycles.

## Introduction

The complex pool of dissolved organic matter (DOM) in the oceans is one of the largest global reservoirs of carbon in the biosphere (662 Pg, Hansell et al., 2009). This DOM pool is almost exclusively accessible to diverse members of the microbial community affecting the biogeochemical state of the ocean and thus the global climate (Azam and Malfatti, 2007). To predict the response of marine ecosystems to natural and anthropogenic perturbations, our understanding on the relation between the organic matter (OM) field and the microbial consortia needs to be refined.

One significant source of OM in the ocean that has been so far largely overlooked are jellyfish (phylum Cnidaria, Class Scyphozoa), which presumably account for >90% of the total global gelatinous zooplankton biomass, estimated to represent 0.038 Pg C in the upper 200 m of the ocean (Lucas et al., 2014). However, due to sampling biases and lack of data, the global jellyfish biomass could be largely underestimated (Lebrato et al., 2019). In fact, jellyfish are ubiquitous and important players in different ecosystems. When conditions are favorable some jellyfish species form extensive blooms, reaching high biomass within a short period of time by efficiently grazing on phytoplankton, zooplankton and fish larvae (Acuña et al., 2011; Pitt et al., 2013). Jellyfish blooms are often short-lived and collapse abruptly due to various largely unexplored environmental factors (Pitt et al., 2014; Schnedler-Meyer et al., 2018; Goldstein and Steiner, 2020). Thus, with their “*boom* and *bust*” population dynamics, jellyfish can represent an important perturbation to the surrounding ecosystem. Despite of the debate over the reported global increase of their blooms and on the actual cause of the observed jellyfish fluctuations, the increase in their population size can have serious socio-economic and ecological consequences (Richardson et al., 2009; Condon et al., 2012; Purcell, 2012; Sanz-Martín et al., 2016). It is undisputed, however, that jellyfish represent a significant source of OM, especially in coastal ecosystems where large blooms are regularly reported (Kogovšek et al., 2010; Lucas et al., 2014).

Yet, the link between jellyfish-derived OM and its most probable consumers and degraders, i.e., the marine microorganisms, has been addressed only by a few studies (Titelman et al., 2006; Condon et al., 2011; Blanchet et al., 2015). Alive jellyfish release copious amounts of colloidal and labile C-rich DOM *via* different mechanisms (i.e., sloppy feeding, fecal pellets and mucus production, and excretion) (Condon et al., 2010, 2011). It has been demonstrated that DOM released by living jellyfish is rapidly respired rather than fueled into the biomass production by otherwise rare members of the ambient microbial community (Condon et al., 2011; Dinasquet et al., 2013; Manzari et al., 2015). However, the significant fraction of the pelagic biomass that jellyfish incorporate during their life span becomes only available to consumers when jellyfish die. Some studies proposed that the OM release after a massive jellyfish die-off should be even higher and of different stoichiometry than the release from living jellyfish (Pitt et al., 2009). Jellyfish consist to >95% of water and the organic content represents between 20 and 40% of their dry weight (Larson, 1986; Lucas et al., 2011; Kogovšek et al., 2014), mainly in the form of proteins (Pitt et al., 2009). This is also reflected by the low molar ratio of carbon to nitrogen (C:N = 4.5:1) of jellyfish biomass (Kogovšek et al., 2014; Molina-Ramírez et al., 2014). Only recently, transcriptome profiling of some jellyfish species provided the baseline allowing first insights into the complexity of this OM pool (Brekhman et al., 2015). Yet, the release rates and the detailed biochemical composition of the OM of different jellyfish taxa remain unknown.

Once jellyfish die, their carcasses start to sink through the water column representing large quantities of jellyfish detrital matter (jelly-OM). Sinking carcasses can be consumed and fragmented by predators and scavengers (Hays et al., 2018), degraded by pelagic microbial communities (Titelman et al., 2006; Tinta et al., 2016) and/or massively deposited at the seafloor (Lebrato et al., 2012), where they can be rapidly degraded by benthic communities (West et al., 2009; Sweetman et al., 2016). Recent estimates on the transfer efficiency of jellyfish biomass indicate that jellyfish are an important component of the biological soft-tissue pump, potentially playing an important role as a food source for the food web of the ocean’s interior (Lebrato et al., 2019). It has been demonstrated that jellyfish carcasses with their proteinaceous character, low C:N ratio and no hard exoskeleton represent a high-quality substrate for specific pelagic bacteria, altering diversity and functioning of marine food web (Titelman et al., 2006; Tinta et al., 2012; Blanchet et al., 2015). Substantial accumulation of NH_4_^+^ and PO_4_^3–^ has been recorded as a result of microbial degradation of jelly-OM in the water column (Tinta et al., 2010; Blanchet et al., 2015). Altogether, this indicates that the pelagic microbial degradation of jelly-OM can be rapid, with important implications for the fate of jelly-OM and biogeochemical cycles in the ocean. However, the key players and the rate and mechanisms of the microbial degradation process of pelagic jelly-OM remain unknown.

Here, we provide a detailed characterization of detrital jelly-OM and further insights into the processing of jelly-OM by microbial communities. To fully understand the nature of the microbial interactions with jelly-OM we have scaled our investigations down to the molecular level, i.e., the scale relevant for microbially mediated biochemical reactions (Azam and Malfatti, 2007). We have characterized the detrital OM of the cosmopolitan coastal meroplanktonic scyphozoan *Aurelia aurita* s.l. on the individual compound level. We link the remineralization rates of different jelly-OM compounds to the metabolic activities of key microbial populations involved in the process. With this approach we ultimately tested the hypothesis that jelly-OM is rapidly degraded by a small, but highly active fraction of the pelagic microbial community.

## Materials and Methods

### Sampling and Pre-processing of Jellyfish Biomass

Specimens of *A. aurita* s.l. were collected in the Gulf of Trieste, northern Adriatic, during the senescent phase of their spring 2018 bloom. Altogether, 27 moribund jellyfish specimens were collected on different days (Supplementary Table S1). The conditions of the collected jellyfish specimens were evaluated based on their activity (i.e., individuals had reduced bell pulsation rate) and on the stage of their body deformation (i.e., individuals had deformed bodies including reduced and/or absent oral arms). Jellyfish were sampled individually from the surface of the water column together with ambient seawater using a large acid-cleaned plastic bucket, rinsed with ambient seawater prior to sampling. After removing the excess ambient seawater, each individual was carefully transferred into a plastic zip-lock bag and stored in a cooling tank in the dark during the transport to the laboratory. Within 1 h after collecting the specimens, jellyfish were stored in the dark at −20°C until further processing.

The collected jellyfish were freeze-dried (at −45°C for 7 d) as proposed elsewhere (Kogovšek et al., 2014) and then weighted to determine the dry weight of each individual (Supplementary Table S1). Next, the jellyfish dry material (jelly-DM) of all 27 individuals was pooled and homogenized with a sterilized pestle and agate mortar. Jelly-DM was then stored in acid- and Milli-Q water-rinsed and combusted glass vials at −20°C until further processing or used in the jellyfish leaching and degradation experiments as described below. To minimize the risk of contamination and degradation of jelly-DM, care was taken to work under sterile conditions, to use combusted glassware and to work on ice at all intermediate steps.

One part of jelly-DM was dialyzed using Spectra/Por 7 Membrane tubing (Sulfur and Heavy Metal Free, Spectrum) with a molecular weight cut-off (MWCO) of 1,000 Da to determine the ratio between the high- (>1,000 Da) and the low- (<1,000 Da) molecular weight compounds (LMW and HMW, respectively) and the C:N ratio of jellyfish organic matter (jelly-OM). The dialysis procedure is described in the Supplementary Material (S Info 1). The LMW fraction of jelly-OM was determined by measuring the concentration of dissolved organic carbon (DOC) and total dissolved nitrogen (TDN) in the dialyzate (Supplementary Table S2). After dialysis, jelly-OM was recovered into an acid- and Milli-Q water-rinsed and combusted glass petri dish and again freeze-dried at −45°C for 7 d. Thereafter, the amount of C and N in the dried jelly-OM material was determined in triplicate after combustion at 1,150°C (Elementar, Vario Micro Cube elemental analyzer) with 3% accuracy.

### Jellyfish Leaching Experiments

The concentration and composition of the particulate (>0.8 μm) and dissolved (<0.8 μm) organic matter (POM and DOM, respectively) and inorganic nutrients leaching from jelly-DM was determined by dissolving 250 mg jelly-DM powder (prepared as described above) in 1 L of artificial seawater (salinity of 35) prepared according to Kester et al. (1967). Jelly-DM was suspended in artificial seawater in an acid- and Milli Q water-rinsed and combusted glass Erlenmeyer flask placed on a shaker in the dark at room temperature. Duplicate experimental flasks were subsampled at 0 h, 30 min and after 1, 2, 6, 8, 12 and 24 h for POC, PN, DOC, and TDN. Samples for total dissolved hydrolyzable amino acids (TDHAA) and inorganic nutrients (see description below) were also collected. At each time point, two technical replicates were collected from each of the two experimental flasks. To check for possible bacterial contamination, samples for bacterial abundance were also collected. No bacterial growth in these incubations was noticed.

### Jellyfish Degradation Experiments

We have conducted two short-term batch culture experiments. For each experiment, six acid-washed, Milli-Q water-rinsed and combusted 5 L (Experiment I) and 10 L (Experiment II) borosilicate glass flasks were filled up with 0.2 μm filtered aged seawater (ASW) and freshly collected 1.2 μm filtered coastal ambient seawater (serving as bacterial inoculum) in a ratio of 9:1. Seawater for both, ASW and the bacterial inoculum was collected at 5 m depth in the center of the Gulf of Trieste (northern Adriatic) using 5 L Niskin bottles connected to a carousel water sampler (SBE 32, Sea-Bird Electronics). Seawater for ASW was collected in August 2018 and aged in acid-washed and Milli-Q water-rinsed 20 L Nalgene carboys for about one month at room temperature in the dark. Seawater for the bacterial inoculum was sampled and filtered on 13 September 2018, the same day as starting Experiment I. Experiment II was set up using the same bacterial inoculum, but kept in the dark at 4°C during Experiment I. Experiment II started immediately after Experiment I was finished.

For each experiment, three of the experimental bottles received 100 mg of jelly-DM L^–1^, representing the jelly-OM treatment. The final concentration of 100 mg of jelly-DM L^–1^ was added to mimic conditions potentially experienced by the ambient microbial community during the decay of *A. aurita* bloom in the Adriatic Sea. There were on average at least 10 jellyfish per m^3^ near the surface of the water column, each having a dry mass of ∼10 g (Supplementary Table S1), which equals to 100 g jelly-DM m^–3^. For each experiment, three experimental bottles were not amended with jelly-OM and served as control. In both experiments, all bottles were incubated in the dark at *in situ* temperature (∼24°C) and mixed thoroughly prior to subsampling. In both experiments, we sampled at short-time intervals and enumerated bacterial abundance instantly after each subsampling. In the Experiment I, we subsampled all the experimental bottles at 0, 6, 12, 24 h and terminated the experiment after 32 h, when the bacterial community reached its late exponential growth phase. In the Experiment II, we subsampled all the bottles at 0, 12, 32, 46, 56, 80 h and terminated the experiment after 84 h when the bacterial community entered its decay phase. For each subsampling about 250 mL was removed from the flasks, leaving therefore about 2/3 of the initial volume in all the experimental bottles at the end of each experiment.

At each time point, subsamples were taken for bacterial abundance, DOC, TDN, TDHAA, dissolved free amino acids (DFAA) and inorganic nutrients from each of the experimental flasks, preserved and analyzed as described below. In addition, the subsample of the bacterial inoculum just prior to the start of both experiments (at 0 h) was taken for bacterial metagenome analyses as described below. Also, subsamples were taken from each of the experimental flasks at the peak of the bacterial abundance (at 32 h, only in Experiment I) and during decay phase of bacterial growth (at 84 h, only in Experiment II) for bacterial metagenome and proteomics analyses as described below. At the same time, subsamples from each of the experimental flasks were incubated with specific fluorogenic reagents to estimate respiration and biomass production of specific bacterial populations (at 0 h in both experiments), at the peak of the bacterial abundance (at 32 h, only in Experiment I) and during the decay phase of bacterial growth (at 84 h, only in Experiment II) as described below. At each subsampling, temperature and the concentration of oxygen were monitored in each of the experimental flasks to check whether the temperature was constant and that the oxygen concentration never dropped below 80% of saturation. A scheme of the jellyfish degradation experiments with subsampling points and analyzed parameters is presented in Supplementary Figure S1.

### Estimating Bacterial Abundance

Two replicates (1.5 mL each) were taken for determining bacterial abundance and fixed with 0.2 μm filtered 37% formaldehyde (2% final concentration). Samples were immediately stored at −80°C until further processing. For enumerating bacteria, 1 mL of sample was filtered onto a 0.2 μm white polycarbonate filter (supported by an 0.45 μm cellulose acetate filter) using a Millipore glass filtration system and a vacuum pump at low pressure (<200 mbar). DAPI-stained (2 μg mL^–1^ in Vectashield) bacterial cells were enumerated using an epifluorescence microscope (Zeiss Axio Imager M2 at 1,250× magnification and the DAPI filter set, Ex/Em = 358/461 nm). The bacterial abundance was calculated based on the average number of cells from at least 20 counting fields with 20–200 cells enumerated per counting field.

### Respiration of Specific Bacterial Populations

The abundance of respiring bacteria was determined at a single-cell level using the Redox Sensor Green reagent (BacLight Redox Sensor Green Vitality Kit, ThermoFisher). This dye results in green fluorescence (Ex/Em = 495/519 nm) when modified by bacterial reductases, many of which are part of electron transport systems and can thus serve as proxy for bacterial respiration (Kalyuzhnaya et al., 2008). A 5 mL subsample of the bacterial inoculum collected in triplicate just prior to the start of each experiment (at 0 h) and 5 mL triplicate subsamples from each of the experimental bottles at the peak of the bacterial abundance (at 32 h, Experiment I) and during decay phase of bacterial growth (at 84 h, Experiment II) were spiked with Redox Sensor Green reagent (RSG) to reach a final concentration of 1 μM. Samples with RSG were incubated in cultivation tubes with vent caps at *in situ* temperature in the dark for 30 min. The bacterial activity was terminated by fixing the sample with 0.2 μm filtered 37% formaldehyde (2% final concentration) and stored at −80°C until further processing. Samples were filtered as described above (see section “Estimating Bacterial Abundance”) and mounted with a DAPI-mix to determine the total abundance of respiring microbial cells by counting individual cells with overlaying DAPI and RSG signal with the DAPI and FITC (Ex/Em = 495/519 nm) filter set, respectively, using a ZEISS Axio Imager 2 microscope at 1,250× magnification. At least 20 fields were counted for each filter slice using the software^1^ ACMEtool2. In parallel, to determine the abundance of specific respiring bacterial populations, filter pieces were processed using the FISH protocol (see section “Fluorescence *in situ* Hybridization”).

### Biomass Production of Specific Bacterial Populations

The biomass production of the bacterial community was determined at the single-cell level based on the incorporation rates of the methionine analog L-homopropargylglycine (HPG) into newly synthesized bacterial proteins (Samo et al., 2014). The incorporation of HPG was detected using click chemistry, where the alkyne-modified HPG is detected with Alexa Fluor 488 azide (Ex/Em = 490/525 nm), following the manufacturer’s protocol (Click-iT HPG Alexa Fluor 488 Protein Synthesis Assay Kit, ThermoFischer). A 5 mL subsample of the bacterial inoculum was collected in triplicate just prior to the start of each experiment and from each of the experimental flasks (in triplicate) at the peak of the bacterial abundance (at 32 h, Experiment I) and during the decay phase of bacterial growth (at 84 h, Experiment II). Subsamples were spiked with 50 μM HPG reagent to reach a final HPG concentration of 20 nM and incubated at *in situ* temperature in the dark in the cultivation tubes with vent cap for 4 h. Bacterial activity was terminated by fixing the sample with 0.2 μm filtered 37% formaldehyde (2% final concentration) and stored at −80°C until further processing. Subsequently, samples were filtered as described above (see section “Estimating Bacterial Abundance”) and filters were further processed according to click reaction protocols as follows: filter slices were incubated in 200 μL of Click-It reaction buffer (154.5 μL Sigma water, 20 μL Click-It reaction buffer, 20 μL 10× reaction buffer additive, 4 μL copper (II) sulfate, 1.6 μL Alexa Fluor 488 azide) in the dark at room temperature for 30 min, followed by a Milli-Q water rinse and air-drying. Afterward, the filter slices were mounted with a DAPI-mix to determine the total abundance of active microbial cells by counting individual cells with overlaying DAPI and HPG signal with the DAPI and FITC (Ex/Em = 495/519 nm) filter set, respectively, using a ZEISS Axio Imager 2 microscope at 1,250× magnification. At least 20 fields were counted for each filter slice using the software ACMEtool2. To determine the abundance of specific HPG incorporating bacterial populations, filter slices were processed using the FISH protocol (see section “Fluorescence *in situ* Hybridization”).

### Fluorescence *in situ* Hybridization

The abundance of bacteria and specific bacterial populations were determined by fluorescence *in situ* hybridization (FISH) using specific oligonucleotide probes labeled with Cy3 at the 5′-end (Biomers) (Supplementary Table S3). The specific bacterial populations were selected based on the relative abundances of the metagenomic assembled genomes (MAGs). An *in silico* analysis was performed to check the coverage and specificity of the probes using SILVA TestProbe 3.0 and the SSU r132 SILVA Database, REFNR sequence collection and 0 as maximum number of mismatches searching for the reverse and complementary sequence of the probe. The specificity of the selected probes for specific bacterial populations of interest was confirmed by blasting sequences of probes against the closest matched genome of each selected MAG. In addition, the specificity of the probes was checked using pure cultures of individual bacterial strains (i.e., a mixed culture of *Alteromonas* isolates (bacterial isolate S2-1-I5P4-O2, S2-2-MA-O1, S3-3-P10-O1, and S1-1-I5P4-O2), a culture of *Vibrio splendidus* (Acc No. JQ432580) and a culture of *Pseudoalteromonas* sp. (Acc No. KC307729) served as positive controls for the Alter2, GV and PSU730 probe, respectively). A sample of *Crenarchaeota*, obtained from an axenic Nitrosopumilus culture (Bayer et al., 2019b) served as negative control. We applied a modified version of the FISH method (Glöckner et al., 1996). For protocol details please see Supplementary Material S Info 2.

Samples were examined with a ZEISS Axio Imager 2 microscope equipped with specific filter sets for DAPI (Ex/Em = 358/461 nm), Cy3 fluorophore (Ex/Em = 554/568 nm) and FITC (Ex/Em = 495/519 nm) at 1,250× magnification. To determine the abundance of respiring bacterial populations, we applied the FISH method to the samples incubated with Redox Sensor Green reagent (see section “Respiration of Specific Bacterial Populations”). The abundance of individual respiring bacteria within specific bacterial populations was determined by counting individual cells with overlaying DAPI, RSG, and FISH signal using DAPI, FITC and Cy3 filter sets, respectively. To determine the abundance of HPG incorporating bacterial populations, we applied the FISH method to the samples incubated with HPG (see section “Biomass Production of Specific Bacterial Populations”). The abundance of individual HPG incorporating bacteria within specific bacterial populations was determined by counting individual cells with overlaying DAPI, HPG, and FISH signals using DAPI, FITC and Cy3 filter sets, respectively. At least 20 fields were counted for each filter slice using the software ACMEtool2.

### Bacterial Metagenomes

Bacterial biomass was collected onto 0.2 μm polyether sulfone membrane filters (PALL Inc.) by filtering 2 L of the (1.2 μm pre-filtered) bacterial inoculum and from each of the triplicate control treatments and 0.5 L from each of the triplicate jellyfish treatments using acid- and Milli-Q water rinsed and combusted filtration sets applying a low (<200 mbar) pressure. Samples from all experimental flasks were taken in Experiment I at 32 h, corresponding to the peak of the bacterial abundance in the jellyfish treatment. Filters were then immediately transferred into sterile cryotubes and stored at −80°C until further processing. Total nucleic acids were extracted from the filters following the protocol of Angel (2012) with some modifications. For details of the extraction protocol please see Supplementary Material S Info 5. We have sequenced the metagenome of the coastal microbiome (by pooling DNA extracted from the bacterial inoculum that we used to set up each of the two experiments) and of the communities from jelly-OM and the control treatments (by pooling DNA extracted from each, the triplicate jelly-OM and the control flasks). All three metagenomic DNA libraries were constructed individually (Westburg kit, enzymatic shearing) and sequenced on one lane of the HiSeqV4 Illumina platform at the Vienna Biocenter Core Facilities^2^. Raw reads were deposited at NCBI under the accession number PRJNA633735. Paired-end reads were assembled from each metagenome with MEGAHIT v.1.1.1 (k list: 21, 29, 39, 59, 79, 99, 119, 141) (Li et al., 2015). Gene prediction was performed with Prodigal under metagenomic mode (-p meta) (Hyatt et al., 2010). For additional information on the metagenomic assembly please see Supplementary Material S Info 5. To obtain an overview of the phylogenetic composition of each metagenome, the phylogenetic affiliation of the predicted genes was identified using the lowest common ancestor algorithm adapted from DIAMOND 0.8.36 blast (Buchfink et al., 2015) by searching against the NCBI non-redundant (NR) database (Sayers et al., 2020). The top 10% hits with an *e*-value <1 × 10^–5^ were used for phylogenetic assignment (–top 10). Reads from each metagenome were mapped to the predicted gene catalog with the BWA algorithm (bwa mem) (0.7.16a) (Li and Durbin, 2009). The gene abundance was estimated by the number of mapped reads and normalized as follows: RPM (mapped reads per million) = 10^6^ × (mapped reads/gene length)/sum of (mapped reads/gene length). For MAG construction, paired-end reads from each metagenome were pooled and co-assembled using MEGAHIT v.1.1.1 (k list: 21, 29, 39, 59, 79, 99, 119, 141). The contigs were clustered with two separate automatic binning algorithms: MaxBin and MetaBAT (2.15) with default settings (Wu et al., 2014; Kang et al., 2015). The generated genomic bins were de-replicated and refined with Metawrap (bin_refinement) (Uritskiy et al., 2018). Bins with >70% completeness and <10% contamination were kept for downstream analysis (-c 70, −x 10). To determine the abundances of the bins across samples, short reads from each metagenome were mapped to the bins using the Metawrap function “quant_bins.”

### Protein Extraction Protocols

#### Extracting Soluble Proteins From the Jellyfish Biomass

For proteomic analyses of jelly-DM, extraction of soluble proteins was performed as follows: 100 mg of jelly-DM was resuspended in lysis buffer (4% SDS, 100 mM Tris-HCl pH 8, EDTA 50 mM pH 8) and incubated at 87°C for 30 min. Cysteines were reduced and alkylated by incubating the suspension with 10 mM dithiothreitol (55°C, for 45 min) and 55 mM iodoacetamide (room temperature, for 1 h). Proteins were precipitated with three volumes of 20% TCA in acetone (final concentration) at −20°C for 4 h and subsequently, washed with ice-cold 100% acetone (Valledor and Weckwerth, 2014). Dried protein pellets were resuspended in 50 mM TEAB buffer (triethyl ammonium bicarbonate buffer, Sigma) and protein concentrations were measured with the Pierce 660 nm Protein Assay Reagent (Thermo Scientific) using BSA (bovine serum albumin) as a standard. Ten μg of proteins of each sample was subjected to in-solution trypsin digestion (1:100, *w*/*w*) at 37°C overnight. Trypsin digestion was terminated by adding trifluoroacetic acid (TFA) to the samples (1% final concentration). Samples were desalted using Pierce C18 Tips (Thermo Scientific) according to manufacturer’s instructions. To increase the number of peptides recruited from our complex matrix (and to identify low-abundant peptides) we used Pierce High pH Reverse-Phase Peptide Fractionation kit (Thermo Scientific) according to manufacturer’s instructions. Prior the LC MS/MS analyses, samples were dissolved in 0.1% formic acid and 2% acetonitrile and transferred into micro-inserts sealed with aluminum caps. Prior to the analyses, peptides were quantified using Pierce quantitative fluorometric peptide assay (Thermo Scientific) according to manufacturer’s protocol. The concentration of peptides ranged from ∼3.3 to ∼43 ng μL^–1^. Thus, a 5 μL injection volume corresponded to 16 to 215 ng of peptides being analyzed in the LC MS/MS (described below).

#### Extracting Soluble Proteins From the Treatments’ Media

We have extracted and sequenced soluble proteins from the jelly-OM treatments by concentrating the fraction of media between 0.2 μm and 5,000 Da. Samples were taken from each of the replicate flasks at the peak of bacterial abundance (at 32 h in Experiment I) and during the decay phase of bacterial growth (at 84 h in Experiment II). Four L of media was filtered through 0.2 μm and concentrated to ∼250 μL in several concentration steps. First, the filtrate (i.e., the media <0.2 μm) was concentrated to 250 mL using a VivaFlow 200 with 30,000 Da Molecular Weight Cut-Off (MWCO) to collect the high molecular fraction (30,000 Da–0.2 μm). This was followed by a further concentration step using a VivaFlow 200 with 5,000 Da MWCO to collect the low molecular fraction (5,000–30,000 Da) at ∼1.75 bar pressure and ∼200 mL min^–1^ flow rate according to manufacturer’s instructions (Sartorius). The high and low molecular fraction were further brought down to 250 μL using an Amicon Ultra-15 Centrifugal Filter 30,000 Da MWCO and 3,000 Da MWCO Unit (Merck-Millipore). NuPAGE sample reducing agent (Invitrogen) was added to samples to reach 1× final concentration. These samples were stored at −20°C until further processing.

Proteins were precipitated with nine volumes of 96% EtOH at −20°C overnight. Pellets were resuspended with 50 mM TEAB buffer (Sigma) and proteins were quantified using Pierce 660 nm Protein Assay Reagent (ThermoFisher). Thereafter, cysteines were reduced and alkylated as described above, followed by another protein precipitation with nine volumes of 96% EtOH at −20°C overnight. Again, pellets were resuspended in 50 mM TAB, followed by overnight in-solution trypsin (Roche) digestion (1:100, *w*/*w*) at 37°C. TFA was added to the samples at 1% final concentration to terminate trypsin digestion. Samples were desalted using Pierce C18 Tips (Thermo Scientific) according to the manufacturer’s protocol. Prior the LC MS/MS analyses, pellets were dissolved in 0.1% formic acid and 2% acetonitrile and transferred into micro-inserts sealed with aluminum caps. Before the run, the concentration of peptides was measured using Pierce Quantitative fluorometric peptide assay (Thermo Scientific). Concentration of peptides ranged from ∼14 to ∼100 ng μL^–1^. Thus, with 5 μL injection volume into the LC MS/MS between 70 and 500 ng of peptides were sequenced.

#### LC-MS/MS Analysis and Peptide Identification

LC-MS/MS analysis and peptide identification were performed as previously described in detail (Bayer et al., 2019a) with slight modifications. For details see Supplementary Material S Info 4. The MS/MS spectra from each proteomic sample was searched using MASCOT engines against the *A. aurita* transcriptome (accession GBRG00000000, Brekhman et al., 2015) and validated with Percolator in Proteome Discoverer 2.1 (Thermo Fisher Scientific) by employing the settings described in Hansen and Koroleff (2007). Briefly, to reduce the probability of false peptide identification, the target–decoy approach was used and results <1% FDR at the peptide level were kept (Elias and Gygi, 2007). A minimum of two peptides and one unique peptide was required for protein identification. Protein quantification was conducted with a chromatographic peak area-based label-free quantitative method (Zhang et al., 2015). The proteomic raw data were deposited at ProteomeXchange under accession number PXD021342 and at jPOST under accession number JPST000960.

### Chemical Analysis

#### Particulate and Dissolved Organic Carbon and Nitrogen

Samples for particulate and dissolved organic carbon (POC and DOC, respectively) and particulate and total dissolved nitrogen (PN and TDN, respectively) were filtered onto combusted Whatman GF/F (∼0.8 μm pore size) filters using acid-, Milli-Q water rinsed and a combusted glass filtration system. GF/F filters were stored at −20°C until analyzed for POC and PN by combustion at 1,150°C with an elemental analyzer (Vario Micro Cube, Elementar) with a 3% accuracy. Approximately 30 mL of the GF/F filtrate was collected into acid-, Milli-Q water rinsed and combusted glass vials and acidified with 12 M HCl (∼100 μL per ∼20 mL of sample) to reach a final pH <2 and stored at 4°C until analysis. DOC and TDN analyses were performed by a high temperature catalytic method using a Shimadzu TOC-L analyzer equipped with a total nitrogen unit (Hansell et al., 1993). The calibration for non-purgeable organic carbon was done with potassium phthalate and for TDN potassium nitrate was used. The results were validated with Deep-Sea Reference (DSR) water for DOC (CRM Program, Hansell Lab). The precision of the method, expressed as RSD% was <2%.

#### Dissolved Inorganic Nutrients

Dissolved inorganic nitrogen (NH_4_^+^, NO_2_^–^, NO_3_^–^) and dissolved inorganic phosphorus (PO_4_^3–^) concentrations were determined spectrophotometrically by segmented flow analysis (QuAAtro, Seal Analytical) following standard methods (Hansen and Koroleff, 2007). The validation and accuracy of the results were checked with reference material (KANSO CO., LTD.) before and after sample analyses. The quality control is performed annually by participating in an intercalibration program (QUASIMEME Laboratory Performance Study).

#### Dissolved Amino Acid Analysis

Samples for total dissolved amino acid analyses were filtered through combusted Whatman GF/F filters using acid-, Milli-Q water rinsed and combusted glass filtration systems. Approximately 4 mL of filtrate was collected in dark glass vial and stored at −20°C until analysis. For each sample two technical replicates were collected. Samples were analyzed for dissolved free amino acids (DFAA) and TDHAA. The concentration of dissolved combined amino acids (DCAA) was calculated as the difference between TDHAA and DFAA. Samples for TDHAA analysis were hydrolyzed as described by Kaiser and Benner (2005) with some modifications (for details see Supplementary Material S Info 3).

For DFAA analysis, 500 μL of sample was directly pipetted into acid-, Milli-Q water rinsed and combusted glass HPLC ampules and analyzed in the same way as total hydrolyzable dissolved amino acids (THDAA) samples. Analysis was performed on a Shimadzu Nexera X2 ultra high-performance liquid chromatograph (UHPLC) with a fluorescence detector (RF-20A XS). Pre-column derivatization was applied with ortho-phthalaldehyde (OPA) according to the protocol of Jones et al. (1981), with slight modifications. For further details on the analysis of dissolved amino acids see Supplementary Material S Info 3.

### Statistical Analysis

Statistical analysis was performed to evaluate the effect of jelly-OM enrichment on chemical and microbiological parameters in our batch experiments using 2-sample *t*-test at 95% confidence interval and assuming homoscedasticity and normality of the data. The statistical difference between measured parameters in triplicate jelly-OM vs triplicate control treatments was analyzed at each time point of each experiment. When possible, i.e., at overlapping time points, the statistical difference between measured parameters in the jelly-OM vs control treatments from the two experiments was analyzed (in this case six replicates from each treatment at overlapping time points were compared). Results were considered significantly different at *p* < 0.05. All statistical analyses were done using VEGAN and STATS package in R Studio R version 3.5.2 (2018-12-20)^3^.

## Results

### Chemical Characterization of Jellyfish Detritus

The jellyfish dry material (jelly-DM) used in our experiments consisted of pooled freeze-dried biomass of 27 *A. aurita* s.l. individuals in moribund state collected in different areas of the Gulf of Trieste in the northern Adriatic Sea during the senescent phase of their spring bloom in the year 2018. The biomass of 27 moribund individuals was pooled to obtain a representative sample of a decaying jellyfish population from the study area. In this way we also avoided possible biases arising from variations in size of different individuals within the population. The rationale behind using freeze-dried material was to preserve its biochemical properties and to ensure homogeneity of the material and thus, reproducibility of the experiments. In this way, our approach ensured that we obtained a representative subsample of a decaying jellyfish biomass, which potentially becomes available to the ambient microbial community in the northern Adriatic Sea, where *A. aurita* regularly forms blooms in the spring – summer period (Kogovšek et al., 2010).

The average dry mass (±SD) of *A. aurita* specimens was 11 ± 4 g (Supplementary Table S1) and the mean carbon (C) and nitrogen (N) content of jellyfish organic matter (i.e., jelly-OM or dry mass of dialyzed material (>1,000 Da), see Supplementary Material S Info 1) was 26.5 ± 2.9% and 6.7 ± 0.7%, respectively, resulting in an average C:N molar ratio of 4.6 ± 0.1.

We performed jellyfish leaching experiments to determine the concentration and composition of the particulate (>0.8 μm) and dissolved (<0.8 μm) organic matter (POM and DOM, respectively) and inorganic nutrients leaching from jelly-DM. The analysis of jelly-POM (>0.8 μm) revealed that on average (±SD) 49 ± 8% of the total organic jelly-C was in the form of particulate organic carbon (POC) and 49 ± 4% of the total jelly-N was particulate organic nitrogen (PON) (Table 1). Thus, the C:N ratio of jelly-POM was 3.2 ± 0.7. The analysis of jelly-DOM fraction revealed that 0.44 ± 0.03 μmol of DOC (mg jelly-DM)^–1^ d^–1^ and 0.13 ± 0.01 μmol of TDN (mg jelly-DM)^–1^ d^–1^ was released into the ambient water, with a C:N ratio of 3.4 ± 0.1:1 (Table 1). The sum of the released POC and DOC fraction represents the total organic carbon (TOC) released from the jelly-DM amounting to 0.86 ± 0.03 μmol (mg jelly-DM)^–1^ d^–1^ or, expressed as weight by weight, 10.35 ± 0.39 μg of TOC (mg jelly-DM)^–1^ d^–1^. Thus, the released TOC represents ∼1% of freeze-dried jelly-DM used in our study, which is in agreement with previous studies (Kogovšek et al., 2014). The jelly-TDN pool consisted to >90% of dissolved organic nitrogen (DON), while the remaining 10% was dissolved inorganic nitrogen (DIN = NH_4_^+^ + NO_3_^–^ + NO_2_^–^) consisted to 70% of NH_4_^+^ (Table 1). In addition, 6.0 ± 0.1 nmol PO_4_^3–^ and 109 nmol of TDHAA were released per mg jelly-DM d^–1^. Approximately 55% of the TDHAA was in the form of dissolved free amino acids (DFAA) (Supplementary Table S4). The most abundant amino acid species in the DFAA pool was glycine, followed by the sulfonic acid taurine, representing 41.9 and 37.8 mol% of the DFAA pool, respectively. Within the jelly-DCAA pool, the most abundant amino acid was Glx (*i.e.*, the sum of glutamic acid and glutamine) with 30.3 mol%, followed by glycine (24.6 mol%), alanine (15.6 mol%) and Asx (*i.e.*, sum of aspartic acid and asparagine) with 11.1 mol% (Supplementary Table S4). All other amino acid species contributed <10 mol% of the jelly-DCAA pool (Supplementary Table S4).

**TABLE 1:** Release rates of POC, PN, DOC, TDN, DIN, DON, NH_4_^+^, NO_3_^–^, NO_2_^–^, and PO_4_^3–^ expressed in μmol released (mg of jelly-DM)^–1^ d^–1^.

	μ mol released (mg of jelly-DM)^–1^ d^–1^	μ g released (mg of jelly-DM)^–1^ d^–1^
POC	0.42 (± 0.07)	5.11 (± 0.84)
PN	0.12 (± 0.01)	1.75 (± 0.14)
DOC	0.44 (± 0.03)	5.24 (± 0.39)
TDN	0.13 (± 0.01)	1.79 (± 0.11)
DON	0.12 (± 0.01)	1.64 (± 0.09)
DIN	0.01 (± 0.01)	0.15 (± 0.12
NH_4_^+^	0.0069 (± 0.0061)	
NO_3_^–^	0.0034 (± 0.0030)	
NO_2_^–^	0.00058 (± 0.0011)	
PO_4_^3–^	0.0063 (± 0.00085)	

*Values are averages (± SD) of four replicates (two technical replicates from the same bottle and of duplicate experiments). DON is calculated as the difference between TDN and DIN. DIN is the sum of NH_4_^+^, NO_3_^–^, and NO_2_^–^. POC, particulate organic carbon; PN, particulate nitrogen; DOC, dissolved organic carbon; TDN, total dissolved nitrogen; DON, dissolved organic nitrogen; DIN, dissolved inorganic nitrogen.*

The dialysis of jelly-DM revealed that most jelly-DOM was composed of high molecular weight compounds (HMW, >1,000 Da), as low molecular weight compounds (LMW, <1,000 Da, *i.e.*, dialyzate) represented only about 6% of the total jelly-DOC and only about 9% of the total jelly-TDN pool (Supplementary Table S2).

We have extracted and identified 10,966 soluble jellyfish proteins from 100 mg of jelly-DM using a proteomics approach (see “Materials and Methods”). Clusters of orthologs groups (COG) functional categories could only be assigned to 26% of the annotated proteins (Supplementary Figure S3). The most abundant proteins with functional categories assigned were associated with posttranslational modification, protein turnover, chaperones (O) and cytoskeleton (Z), followed by proteins associated with signal transduction mechanisms (T), translation, ribosomal structure and biogenesis (J), and proteins with unknown function (S) (Supplementary Figure S3). However, by blasting against the NR database, 6978 (or 74% proteins of all retrieved protein sequences) could be annotated and 2,820 unique proteins assigned (Supplementary Table S5). Among these, the most abundant hits were fibrillin-like (8%), myosin-like (7%), actin-like (3%), ubiquitin-like (3%), ribosomal (3%), and collagen-like (1%) proteins (Supplementary Table S5).

### Microbial Consortia Degrading Jellyfish Detritus

**FIGURE 1:**
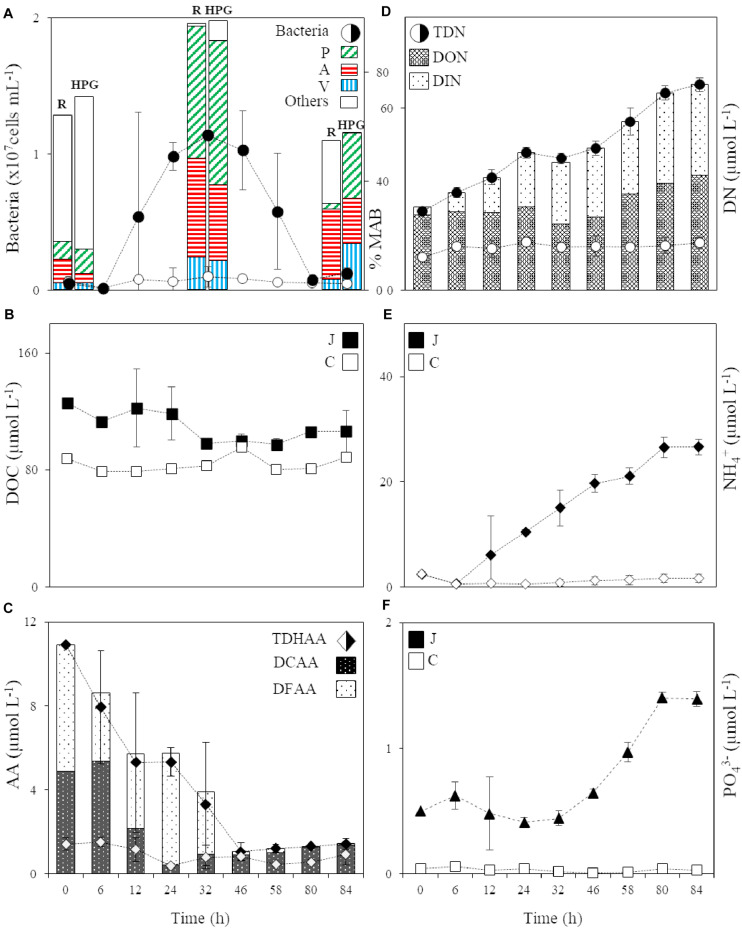
**(A)** Abundance of the bacterial community in the jelly-OM (filled circle) and control (circle) treatment and the percentage of respiring (R) and HPG incorporating (HPG) bacteria in the two jellyfish degradation experiments (average of both experiments ± SD), together with the percentage of respiring and HPG incorporating *Pseudoalteromonas* (P), *Alteromonas* (A), and *Vibrio* (V) and other bacterial populations (Others) within the active microbial community at T0, in the late exponential (at 32 h, Experiment I) and decay phase of bacterial growth (at 84 h, Experiment II), MAB stands for Metabolically Active Bacteria; **(B)** DOC in the jelly-OM (filled square) and control (square) treatments; **(C)** TDHAA, DCAA and DFAA in the jelly-OM (filled diamond) and TDHAA in control (diamond) treatments; **(D)** TDN, DON and DIN in the jelly-OM (filled circle) and TDN in control (circle) treatments; **(E)** NH_4_^+^ in the jelly-OM (filled diamond) and control (diamond) treatments; **(F)** PO_4_^3–^ in the jelly-OM (filled triangle) and control (triangle) treatments in the two jellyfish degradation experiments (average of both experiments ± SD). In panels **(B, E, F)** J stands for jelly-OM treatment and C stands for control treatment without jelly-OM amendment.

Two short-term microcosm experiments were conducted to follow the microbial degradation of jellyfish detritus and the dynamics of the jellyfish-degrading microbial community over time. Based on the long-term monitoring of *A. aurita* populations in the northern Adriatic (T. Kogovšek and A. Malej, unpublished data) and the average dry mass of collected moribund jellyfish, we estimated that during the decay of the bloom the ambient marine microbiota experiences about 100 mg of jelly-DM L^–1^ (see section “Materials and Methods” for details on the setup of the jellyfish degradation experiment). The addition of 100 mg jelly-DM L^–1^ in the jelly-OM’s treatments resulted in an enrichment of 38.4 ± 10.7 μmol L^–1^ of DOC and 15.9 ± 0.5 μmol L^–1^ of TDN (Figures 1B,D), which fits remarkably well with the results from our jelly-DM leaching experiment (Table 1).

**FIGURE 2:**
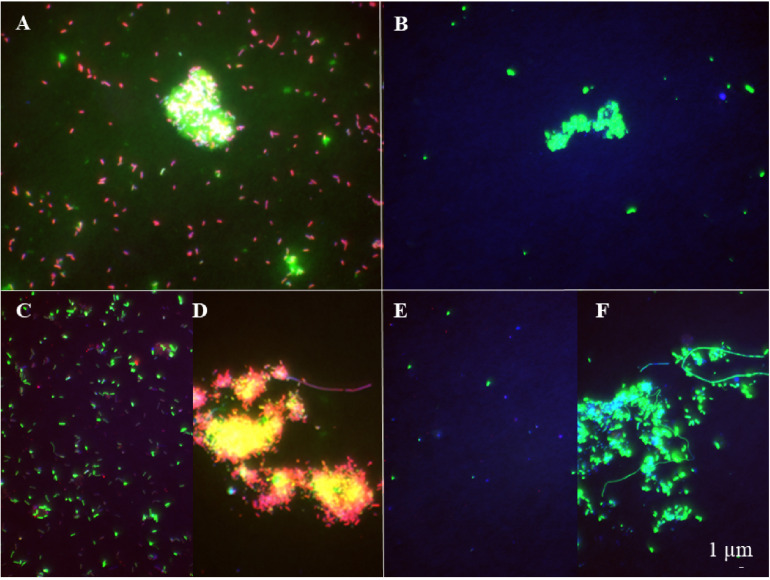
Epifluorescence microscopy images of respiring **(A, B)** and bacterial populations taking up HPG **(C–F)** at the late exponential phase **(A, C, D)** and the senescent phase **(B, E, F)** in the jelly-OM treatment. Red colored cells – bacteria hybridized with FISH probe and stained with DAPI, green colored cells – bacteria hybridized with FISH probe, stained with DAPI and incorporating HPG **(C–F)** and Redox Sensor Green **(A, B)**.

The abundance of the microbial community in the jelly-OM treatments was at the beginning of the incubation experiment 5.7 ± 1.1 × 10^5^ cells mL^–1^, with 54 ± 14% of the bacteria respiring, as determined by the Redox Sensor Green approach combined with FISH (Figures 1A, 2A,B). Using the methionine analog HPG and click chemistry coupled with the FISH method, 48 ± 23% of the bacteria were taking up HPG (Figures 1A, 2C–F).

Based on taxonomic profiling of metagenomic data, the coastal microbiome was diverse (Supplementary Table S6) and dominated by Alphaproteobacteria (61% of bacteria classified at class level, mostly *Pelagibacteriaceae*), followed by Gammaproteobacteria (27%, mostly *Alteromonadaceae*) and *Flavobacteria* (5%) (Supplementary Figure S4). This ambient microbial community responded rapidly to the jelly-OM amendment in both experiments. The average community growth rate based on the increase in cell abundance over time during exponential growth was 0.09 ± 0.01 h^–1^ (Table 2). The microbial community (98 ± 18% were bacteria) reached its peak abundance already after 12 and 32 h in the jelly-OM treatment in Exp I and Exp II, respectively (Figure 1A and Supplementary Table S7).

**TABLE 2:** Bacterial community growth parameters (average ± SD) of three biological replicates per treatment (jelly-OM vs control) of each of the two jelly-OM degradation experiments (Experiment I and Experiment II) and of both experiments combined (average ± SD) of six biological replicates per treatment (jelly-OM vs control) with *p*-values (Student’s *t*-test).

	Experiment I		Experiment II		Average		
	Control	Jelly-OM	Control	Jelly-OM	Control	Jelly-OM	*p*-values
BCD (ΔDOC) (μg C L^–1^h^–1^)	1.5 ± 1.4	27 ± 25	8 ± 5	23 ± 4	4.7 ± 4.6	24.8 ± 16.1	0.015
BB (μg C L^–1^)	14 ± 5	232 ± 16	26 ± 6	210 ± 51	19.8 ± 8.2	221.2 ± 35.7	0.0000001
μ (h^–1^)	0.04 ± 0.01	0.1 ± 0.01	0.005 ± 0.008	0.09 ± 0.02	0.02 ± 0.02	0.09 ± 0.01	0.00009
BP (μg C L^–1^h^–1^)	0.4 ± 0.2	11 ± 1	0.2 ± 0.4	16 ± 5	0.3 ± 0.2	13.6 ± 3.9	0.00001
BR (μg C L^–1^h^–1^)	1.3 ± 1.2	17 ± 24	8 ± 5	7 ± 2	4.5 ± 4.6	11.9 ± 16.1	0.3
BGE (%)	8 ± 7	80 ± 73	1 ± 6	70 ± 11	4.6 ± 6.9	65 ± 27	0.0003

*BCD, bacterial carbon demand; BB, maximum bacterial biomass reached; μ, growth rate; BP, bacterial production; BR, bacterial respiration; BGE, bacterial growth efficiency.*

The jelly-OM supported growth of the bacteria resulting in a less diverse community than in the control treatment and in the coastal microbiome, composed mainly of Gammaproteobacteria (>89%), with *Pseudoalteromonadaceae*, *Alteromonadaceae*, and *Vibrionaceae* accounting for ∼86% of all Gammaproteobacteria, as taxonomic profiling of metagenomic data revealed (Supplementary Figure S4). The abundance and growth rates of the community in the unamended control flasks were significantly lower than in the jelly-OM treatment in both experiments (*t*-test: *p* < 0.001 and *p* < 0.01 in Exp I and Exp II, respectively) (Table 2 and Supplementary Table S7). The community growing in the control flasks was composed mainly of Gammaproteobacteria (64%), but also a considerable fraction of bacteria was affiliated with Alphaproteobacteria (28%) (Supplementary Figure S4).

We constructed 84 MAGs from the metagenomic co-assembly (of the coastal microbiome and the communities of the jelly-OM and control treatment). By mapping the reads back to the MAGs, we found that the relative abundance (the percentage is based on the RPM of specific MAG divided by the sum of RPM of all MAGs, i.e., RPM-based abundance) of some of the 84 MAGs increased in the jelly-OM treatment, mainly due to MAGs affiliated with Gammaproteobacteria (Supplementary Figure S5). We identified *Alteromonas* (with a single genomic bin representing 43% of all MAGs in the jelly-OM treatment), *Pseudoalteromonas* (with two MAGs, representing together 39% of all MAGs in the jelly-OM treatment) and *Vibrio* (with a single genomic bin representing 7% of all MAGs in the jelly-OM treatment) as major jelly-OM degraders (Supplementary Figure S5). These dominant MAGs together represented 88% of all MAGs at the peak of the bacterial abundance in the jelly-OM treatment. At the same time, the sum of their relative abundance represented <15% of MAGs in the control treatment and <0.2% of the MAGs of the coastal microbiome used as an initial inoculum, in agreement with the taxonomic profile of the metagenomic data (Supplementary Figures S4, S5).

The taxonomic profiling of the metagenomes resembled the abundance data obtained by the microscopy-based FISH analysis. At the peak of the bacterial abundance in the late exponential phase, 50 ± 16% of the bacteria in the jelly-OM treatment were identified as *Pseudoalteromonas*, 31 ± 10% as *Alteromonas* and 11 ± 2% as *Vibrio*, together representing >90% of all bacteria detected (Supplementary Table S7). The abundance of *Pseudoalteromonas*, *Alteromonas*, and *Vibrio* populations was significantly lower in the control than in the jelly-OM treatment (*t*-test: *p* < 0.01, *p* < 0.001, and *p* < 0.001, respectively) and in the coastal microbiome they represented only 9 ± 4%, 8 ± 0.7%, and 3 ± 1.7%, respectively, of all bacteria (Supplementary Table S7). Thus, in the jelly-OM treatment the populations of *Pseudoalteromonas*, *Alteromonas*, and *Vibrio* increased 70-, 77-, and 100-fold in their absolute abundance, respectively compared to their contribution in the initial inoculum.

### Microbial Processing of the Jellyfish DOC Pool

During the exponential growth of bacteria in the batch cultures amended with jelly-DM DOC decreased by 24.8 ± 16.1 μg C L^–1^ h^–1^ (Table 2). Assuming that the bacterial metabolism was only fueled by DOC, the decrease in DOC resembles the heterotrophic bacterial carbon demand (BCD). The BCD represents the sum of the amount of carbon consumed for the synthesis of new bacterial biomass, i.e., bacterial production, and the amount of carbon respired. From the increase of bacterial biomass (assuming a C-content of 19.8 fg C cell^–1^, Lee and Fuhrman, 1987) during the exponential growth phase, a bacterial production of 13.6 ± 3.9 μg C L^–1^ h^–1^ was calculated (Table 2). The difference between the BCD and the bacterial C-biomass production is essentially bacterial respiration amounting to 11.1 ± 16.9 μg C L^–1^ h^–1^ (Table 2), based on six biological replicates of the jelly-OM treatment from both experiments. While bacterial production was significantly higher in the jelly-OM than in the control treatment (*t*-test: *p* < 0.0001), there was no significant difference in bacterial respiration between jelly-OM and the control treatment (Table 2). The similar respiration rate in the jelly-OM and the control treatment, however, might be caused by the variability in DOC concentrations in the biological replicates in the jelly-OM in Experiment I (Supplementary Table S9). Likewise, the high variability of the respiration rates in the jelly-OM treatments is likely due to the heterogeneity of the jelly-DOC pool among the six biological replicates. The bulk bacterial growth efficiency (BGE) calculated from the increase in bacterial abundance converted to biomass production and the decrease in DOC concentration was 65 ± 27% in the jelly-OM and 4.6 ± 6.9% in the control treatment (*t*-test: *p* < 0.001, Table 2).

To determine the abundance of respiring bacteria, we combined the redox dye Redox Sensor Green as an indicator of bacterial reductase activity with the FISH approach (Figures 2A,B). To determine the abundance of biomass producing bacteria, we determined the incorporation of the methionine analog, HPG into bacterial proteins, combining click chemistry and FISH (Figures 2C–F). At the peak of the bacterial abundance in the jelly-OM treatments, 98 ± 12% of bacteria were respiring, with *Pseudoalteromonas* representing 50 ± 6%, *Alteromonas* 37 ± 4%, and *Vibrio* 12 ± 1% of the respiring bacterial community (Figures 1A, 2A and Supplementary Table S8). At the same time, 99 ± 29% of the bacteria incorporated HGP, with *Pseudoalteromonas* contributing 53 ± 14%, *Alteromonas* 28 ± 7%, and *Vibrio* 11 ± 3% to the HPG incorporating bacteria (Figures 1A, 2C,D and Supplementary Table S8).

After the bacterial community entered its stationary phase at ∼46 h, the microbial abundance decreased to 1.2 ± 0.4 × 10^6^ cells mL^–1^ until the end of the experiment (at 84 h) with 71 ± 7% identified as bacteria using FISH (Figure 1A and Supplementary Table S7). Even at the end of the batch culture incubations, the abundance of metabolically active bacteria was still significantly higher in the jelly-OM than in the control treatment (*t*-test: *p* < 0.05 and *p* < 0.01 for respiring and biomass producing bacteria, respectively), except for *Alteromonas*, which was equally contributing to the respiring population in both treatments (Supplementary Table S8). In the senescent phase, most bacteria were aggregated and the composition of the metabolically active bacterial community shifted. The respiring population of *Pseudoalteromonas* decreased to 4 ± 1% of the respiring bacterial community and *Vibrio* to 6 ± 2%, while *Alteromonas* represented almost half of the respiring community (47 ± 12%) (Figures 1A, 2B and Supplementary Table S8). Therefore, other microbial populations not targeted with the FISH probes were likely contributing to the respiring bacterial community.

In contrast to the respiring bacterial community, the HPG incorporating bacteria in the senescent phase were still dominated by *Pseudoalteromonas* (41 ± 0.2%), *Alteromonas* (29 ± 0.1%), and *Vibrio* (29 ± 0.1%) in the jelly-OM treatment (Figures 1A, 2E,F and Supplementary Table S8). The rapid decay of bacterial populations in the senescent phase of the batch cultures was probably caused by viral and/or protist grazing, as virus-like particles reached twice the bacterial abundance shortly after its peak at ∼46 h (Supplementary Figure S6A). At the same time, an increase of respiring protists was observed in the jelly-OM treatments (personal observation, Supplementary Figure S6B). As the bacterial community entered its senescence phase, the concentration of DOC in the jelly-OM treatment decreased and fluctuated only slightly until the end of the experiment after 84 h to concentrations similar to that of the control treatments (Figure 1B). The abundances of respiring and HPG incorporating bacterial populations were significantly lower in the control than in the jelly-OM treatments throughout the experiment (*t*-test: *p* < 0.001 and *p* < 0.01 for respiring and biomass producing bacteria in the late exponential phase, respectively, and *p* < 0.05 and *p* < 0.01 for respiring and biomass producing bacteria in the decay phase, respectively; Supplementary Table S8).

### Microbial Processing of Jellyfish Proteins

**FIGURE 3:**
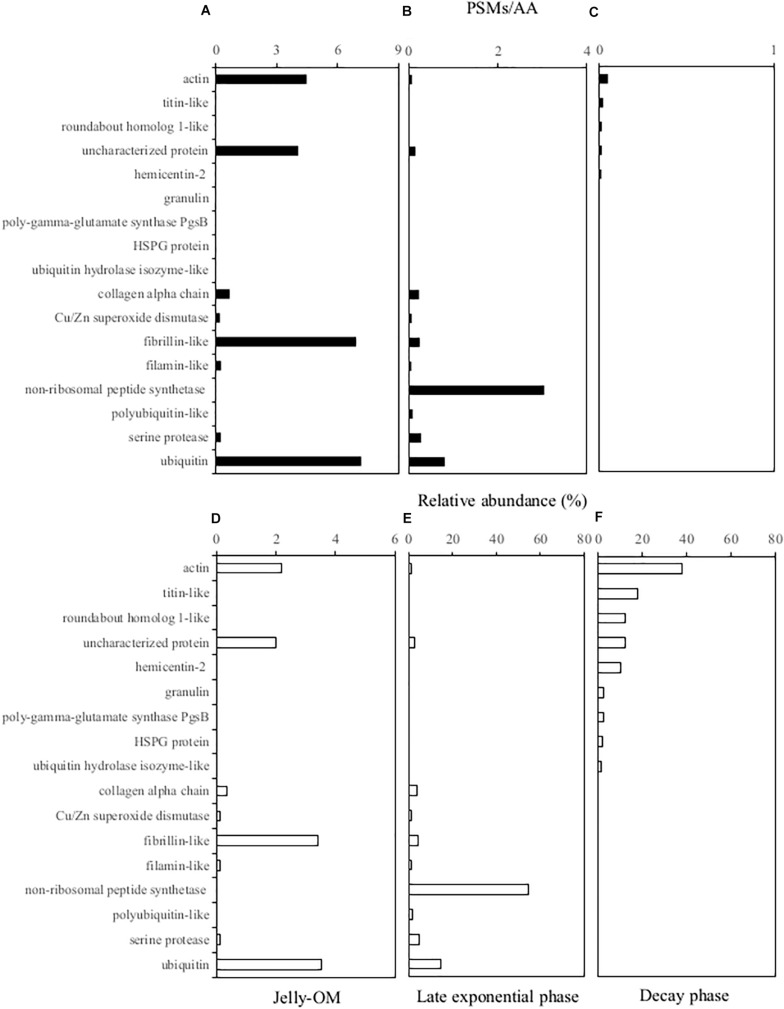
Soluble jellyfish proteins **(A, D)** detected in the 0.2 μm–5,000 Da fraction of the media at the beginning of the experiment in the jelly-OM treatments **(B, E)** in the late exponential phase and **(C, F)** in the senescent phase of the bacterial growth. For each peptide the absolute peptide spectrum matches/number of amino acid residuals (PSMs/AA) value is provided **(A–C)** and its relative abundance **(D–F)** is calculated as the percentage of all the jellyfish proteins detected in the initial jelly-OM **(A)** and at the given time point **(B, C)**. Only peptides >1% of relative abundance at any given time point are presented.

The soluble proteins were extracted from the 0.2 μm – 5000 Da protein fraction of the jelly-OM treatments at the peak of the bacterial abundance (at the end of the Experiment I, at 32 h) and at the senescent phase of bacterial growth (at the end of the Experiment II, at 84 h). We used the peptide spectrum matches (PSMs) divided by the number of amino acid residuals as a proxy for the absolute abundance of peptides (i.e., PSMs/AAs). By summing up the PSMs/AAs values of all the jellyfish proteins detected at a given time point, we followed the changes in the jellyfish protein abundance throughout the experiments (Figure 3).

The obtained protein sequences screened against the *A. aurita* transcriptome (Brekhman et al., 2015) indicated that at the end of the exponential growth bacteria had consumed >97% of the soluble jellyfish proteins detected in the initial jellyfish protein pool (calculated from the decrease of the sum of the PSM/AAs of all the jellyfish proteins from 203.45 at the beginning the experiment to 5.56 at 32 h, Supplementary Table S5). The soluble jellyfish proteins still detectable in the media in the late exponential phase of bacterial growth mainly consisted of non-ribosomal peptide synthetases (>54%), followed by ubiquitin-like proteins (>14%), serine protease (5%), fibrillin-like (4%), collagen alpha chain-like proteins (4%), polyubiquitin-like (2%), actin-like proteins (1%), uncharacterized proteins (3%) and others (<1% of relative abundance) (Figure 3 and Supplementary Table S5). By the time when bacteria entered the senescent phase, <0.1% of jelly-OM proteins were left in the media (calculated from the decrease of the sum of the PSM/AAs of all the jellyfish proteins from 203.45 at the beginning of the experiment to 0.13 at 84 h, Supplementary Table S5). The remaining protein pool was mainly composed of actin-like proteins (38%), titin-like proteins (18%), roundabout homolog 1-like (12%), hemicentin-like (10%), granulin (3%), mesoglein (1%), uncharacterized proteins (12%), and some others (mostly <1% in relative abundance) (Figure 3 and Supplementary Table S5).

### Microbial Processing of the Jellyfish Amino Acid Pool

About 90% of jelly-DON was identified as DFAA and DCAA (Table 1 and Supplementary Table S4). By adding 100 mg of jelly-OM L^–1^, we supplied the ambient bacterial community with about 8-times higher concentrations of TDHAA than present in the northern Adriatic seawater (Figure 1C). In contrast to the jelly-TDHAA pool consisting to 55% of DFAA, the northern Adriatic TDHAA pool was mainly composed of DCAA (88 ± 10%) (Figure 1C and Supplementary Figure S2B). Despite the overall TDN accumulation, jelly-TDHAA decreased in the jelly-OM treatments during the incubation experiment (Figure 1C). By the end of the exponential growth phase, bacterial net consumption of the jelly-AA pool amounted to 70% of the initial pool, corresponding to a net uptake rate of 186 ± 9 nmol L^–1^ h^–1^ (Figure 1C and Supplementary Figure S2). Initially, bacteria consumed mainly the DFAA fraction, decreasing the jelly-DFAA pool by 46 ± 13% within the first 6 h. When the bacterial community entered the stationary phase (∼46 h), 97 ± 1% of the originally available jelly-DFAA pool was consumed, corresponding to a net consumption rate of 103 ± 9 nmol L^–1^ h^–1^ (Figure 1C). During the exponential growth, bacteria consumed more than 99% of the most abundant jelly-DFAA species (Supplementary Figure S2). Glycine was consumed at a rate of 52.1 ± 0.1 nmol L^–1^ h^–1^ and taurine at the rate of 47.1 ± 0.01 nmol L^–1^ h^–1^ (Supplementary Figure S2). At the same time, however, an accumulation of some DFAA species was detected, probably resulting from bacterial degradation of jellyfish proteins. Leucine accumulated at a net rate of 148 ± 9 nmol L^–1^ h^–1^, reaching a concentration of 3.9 ± 0.2 μmol L^–1^ after 32 h (Supplementary Figure S2). After the DFAA pool was depleted, the DCAA pool was consumed by the bacterial community at a net rate of 152 ± 37 nmol L^–1^ h^–1^, leaving only 15 ± 2% of the original jelly-DCAA after the first 24 h when bacteria entered late exponential phase (Figure 1C and Supplementary Figure S2). The concentration of DFAA and DCAA was significantly lower in the control than in the jelly-OM treatments during the late exponential phase of bacterial growth (*t*-test: *p* < 0.001 and *p* < 0.05 for Exp I, *p* < 0.05 and *p* < 0.01 for Exp II).

When the bacterial community entered the senescent phase, the concentration of amino acids started to increase slightly with a net rate of 10 ± 7 nmol L^–1^ h^–1^ due to the increase in DCAA concentrations at a net rate of 13 ± 7 nmol L^–1^ h^–1^ (Figure 1C and Supplementary Figure S2). At the same time, the DFAA pool decreased at a net rate of 3.1 ± 0.5 nmol L^–1^ h^–1^ (Figure 1C and Supplementary Figure S2). At the end of the experiment (from 80 to 84 h), between 1.3 ± 0.1 and 1.4 ± 0.3 μmol L^–1^ or about 13% of the initial jelly-TDHAA pool was left in the media, which is significantly higher than the 0.5 ± 0.1 μmol L^–1^ TDHAA concentrations measured in the control treatment after 80 h (*t*-test: *p* < 0.01) and slightly higher than the concentrations measured in the seawater prior to the experiment (1 μmol L^–1^) (Figure 1C). In the jelly-OM treatment, the remaining amino acid pool consisted of DCAA (96 ± 2%) and dissolved free tryptophan, which was gradually increasing from the stationary to the senescent phase of bacterial growth with a net accumulation rate of 3.8 ± 0.5 nmol L^–1^ h^–1^, representing 86 ± 3% of the DFAA pool at the end of the experiment (Supplementary Figure S2).

### Microbial Processing of the Jelly-TDN and -P Pool

The jelly-TDN pool was composed to >90% of DON (Table 1). In the jelly-OM treatments, the DOC:DON ratio increased from 3.4 ± 0.1 at the start of the experiments to 5.5 ± 0.6 during bacterial exponential growth (Supplementary Figure S7). Jelly-DON was consumed at a net rate of 0.12 ± 0.01 μg N L^–1^ h^–1^ during this exponential growth phase (Figure 1D). At the same time, DIN accumulated in the media at a net rate of 0.52 ± 0.05 μg N L^–1^ h^–1^ in the jelly-OM treatment (Figure 1D). Until the end of the exponential growth phase (at 32 h), the average net accumulation rate of TDN was 0.58 ± 0.05 μmol TDN L^–1^ h^–1^ (Figure 1D). The TDN continued to accumulate even when the bacterial community entered the senescent phase with a net accumulation rate of 0.51 ± 0.05 μmol TDN L^–1^ h^–1^, reaching 67.7 ± 2.3 μmol TDN L^–1^ at the end of the experiment (after 84 h). At the end of the experiment, the concentration of DON and DIN was approximately equal. DON accumulated with a net rate of 0.39 ± 0.08 μmol L^–1^ h^–1^, while the net accumulation of DIN was largely due to NH_4_^+^ accumulating at a net rate of 0.29 ± 0.02 μmol NH_4_^+^ L^–1^ h^–1^, accounting for 80% of the total DIN pool at the end of the experiment in the jelly-OM treatments (Figure 1E).

Not only NH_4_^+^, but also PO_4_^3–^ accumulated in the jelly-OM treatments at a net rate of 0.016 ± 0.001 μmol PO_4_^3–^ L^–1^ h^–1^. Toward the end of the experiment, when bacteria entered the senescent phase, 1.4 ± 0.1 μmol PO_4_^3–^ L^–1^ was measured in jelly-OM treatments (Figure 1F). The TDN concentration and PO_4_^3–^ were significantly lower and changed only slightly in the control treatments (*t*-test: *p* < 0.0001, Figures 1D–F).

## Discussion

### Jellyfish in the Framework of the Ocean’s Detrital Pool

Most studies on the utilization and degradation of detrital particles have focused on detritus of (micro)algae, crustacean zooplankton and appendicularians (Anderson et al., 2017). However, jellyfish detritus represents a substantially fraction of the marine detrital pool, particularly at a regional scale during the decay of massive bloom events, especially in coastal marine ecosystems (Lucas et al., 2014; Lebrato et al., 2019).

In this study, we provide a comprehensive characterization of detritus of a cosmopolitan coastal bloom-forming jellyfish, the meroplanktonic scyphozoan *A. aurita* s.l. Based on the C:N ratio, the pool of detrital OM used in our study was well within the range reported for the populations present in the Adriatic Sea (Kogovšek et al., 2014) and slightly higher than the average values reported for the order *Semaeostomeae* (Cnidaria, Scyphozoa) (3.75 ± 0.31, Lucas et al., 2011). Hence, the jelly-OM we used in this study is representative for a *A. aurita* population. Unfortunately, there are no data on the relationship between the C:N ratio, biochemical composition and different health condition of mature medusae (e.g., healthy individuals vs. moribund). Hence, it is difficult to evaluate whether the jelly-OM used in our study is in fact representative of a decaying jellyfish population. Nevertheless, from what is known it seems that the content of proteins, carbohydrates, free amino acids and OM as a whole gradually decreases with an increase in size and thus possibly with maturity of individuals (Lucas, 1994; Anninsky, 2009).

Our analysis revealed that about half of the OM in the jellyfish detritus is rapidly leaching into the ambient water and thus, available as DOM (<0.8 μm) and consequently, exclusively and readily accessible to microbes. This has important implications for the cycling and fate of this OM pool in the ocean and implies that a considerable fraction of this jelly-OM is utilized in the water column. Larger jellyfish detrital particles are accessible to large organisms [*i.e.*, scavengers and zooplankton (Giering et al., 2014)] and subjected to physical forces fragmenting the jelly-POM into slow-sinking particles. Our data indicate, however, that about half of jellyfish detrital matter, its DOM fraction, might be consumed and degraded solely by pelagic microbial communities (Tables 1, 2). We also found that low molecular weight jelly-DOM (<1,000 Da) represents <10% of the jelly-DOM pool, implying that most jelly-DOM is composed of complex polymeric compounds (Supplementary Table S2).

The low C:N ratio of jelly-OM is indicative of its proteinaceous character, in agreement with previous studies reporting that jelly-OM is composed mostly of proteins (70 ± 14%), followed by lipids (22 ± 12%) and carbohydrates (7 ± 5%) (Anninsky, 2009; Pitt et al., 2009; Merquiol et al., 2019). Our detailed analysis of the soluble protein pool of jellyfish detritus revealed that it is composed mostly of proteins associated with elasticity (fibrillin-like), muscle contraction (myosin-, actin-like), structural proteins (collagen-like), but also by many others, in line with the *A. aurita* transcriptome profile of adult stage medusae (Brekhman et al., 2015). In contrast to jellyfish, fresh detritus of phytoplankton origin has a C:N ratio of ∼6.6 (Redfield et al., 1963) and is on average composed to 40 ± 7% of proteins, 26 ± 14% of carbohydrates, and 15 ± 8% of lipids (Rios et al., 1998). The C:N ratio of crustacean zooplankton varies between 4.8 and 6.2, with proteins accounting from 20 to 70%, lipids from 0.5 to 74% and free amino acids, chitin and carbohydrates between 2 and 10% of dry weight (Ventura, 2006). Hence, in contrast to crustacean zooplankton, jellyfish have, on average, 50% less lipids and lack a chitinous exoskeleton (Pitt et al., 2013). Altogether, this indicates that jellyfish detritus differs substantially from detritus of both phytoplankton and crustacean zooplankton origin. Furthermore, the composition of jellyfish detritus implies that it represents a high quality and easily degradable substrate for heterotrophic marine bacteria (Benner, 2002) that could become available to ambient water microbial communities in large quantities at the demise of jellyfish blooms.

We have simulated the scenario potentially experienced by coastal pelagic microbial communities after a decay of a jellyfish bloom under controlled laboratory conditions. This approach allowed us not only to follow the response of a coastal microbiome to this specific type of detrital material, but also to attribute the recorded degradation/remineralization rates of jellyfish-compounds to metabolic activities of key microbial populations involved in this process. During a typical bloom of *A. aurita* in the coastal northern Adriatic there are on average 10 individuals per m^3^, with each having a dry mass of ∼ 10 g. This would mean an enrichment of about 100 g of jelly-DM m^–3^. If we assume 2% of C for freeze-dried material (Kogovšek et al., 2014), this means an enrichment of 2 g organic C m^–3^, which is well within the range reported for coastal ecosystems globally (Lucas et al., 2014).

Our results show that during a decay of a typical *A. aurita* bloom in the northern Adriatic Sea, when approximately 100 mg of jelly-DM L^–1^ are released, ∼44 μmol L^–1^ of DOC, 13 μmol L^–1^ of TDN (mostly DON compounds), 11 μmol L^–1^ of THDAA (∼55% as DFAA with a considerable amount of free glycine and taurine) and a substantial amount of PO_4_^3–^ (0.6 μmol L^–1^) becomes potentially accessible to the coastal marine microbiome. This significant pulse of labile DOM, with a C:N ratio of 3.4 ± 0.1, and inorganic nutrients represents an important perturbation for pelagic microbial communities, in particular in oligotrophic and/or P-limited marine systems like, *e.g.*, the northern Adriatic Sea (Mozetič et al., 2010; Klun et al., 2019).

### Jellyfish Detritus Is Rapidly Degraded by a Simple Consortium of Opportunistic Bacteria

The addition of jellyfish detrital matter supported rapid growth of the bacterial community with growth rates of ∼2 d^–1^, which are similar or higher than previously reported (Titelman et al., 2006; Tinta et al., 2012; Blanchet et al., 2015). These growth rates are considerably higher than global marine bacterial community growth rates reported for the epipelagic ocean (0.1–1 d^–1^, Ducklow and Kirchman, 2000).

However, not all bacteria thrived under these conditions. It appears that the jellyfish-degrading consortium is composed of specific opportunistic bacterial populations. A rapid shift (within 1.5 d) was observed in the bacterial community from a diverse coastal assemblage dominated by Alphaproteobacteria (resembling a typical assemblage for the region, Tinta et al., 2015) to a community of low diversity composed mainly of Gammaproteobacteria, with *Pseudoalteromonadaceae*, *Alteromonadaceae*, and *Vibrionaceae* accounting for ∼86% of all Gammaproteobacteria (Supplementary Figure S4 and Supplementary Table S6). The observed structural shift is in accordance with previous studies, consistently reporting a dramatic decrease of Alphaproteobacteria and a rapid increase of Gammaproteobacteria growing on fresh and labile jellyfish detritus followed by a succession of Bacteroidetes growing on more complex and presumably less-labile jellyfish OM (Tinta et al., 2012; Dinasquet et al., 2013; Blanchet et al., 2015).

By coupling taxonomic profiling of our metagenomic data with microscopy-based tracking of individual metabolically active bacterial populations of predominant MAGs, we show that the DOM fraction of *A. aurita* detritus can be degraded by a simple consortium composed of only three dominant gammaproteobacterial populations, *Pseudoalteromonas*, *Alteromonas*, and *Vibrio* (Supplementary Figure S4). This suggests that an abundant source of high quality and bioavailable DOM reduces the biodiversity of bacteria by favoring a small number of copiotrophs dominating the community (Kolmakova et al., 2019). These opportunistic populations accounted for >90% of all metabolically active (both respiring and HPG incorporating) bacteria in the jellyfish-degrading community and rapidly consumed almost the entire pool of jellyfish proteins (>98%), amino acids (∼70%) and jelly-DOC within ∼1.5 d, indicating a rapid turnover of jellyfish-DOM, including soluble proteins (Figures 1, 3 and Supplementary Figure S5, and Supplementary Table S5).

### Bacteria Growing on Jellyfish-DOM Exhibit High Growth Efficiency

The simple bacterial consortium growing on jelly-OM exhibited a growth efficiency of 65 ± 27%, calculated based on the increase in abundance of bacteria during their exponential growth and the concurrent decrease in jelly-DOC (Figure 1, Table 2, and Supplementary Table S8). This growth efficiency greatly exceeds the bulk growth efficiency of oceanic surface water bacteria (15 ± 12%) and coastal bacterioplankton (27 ± 18%) areas (del Giorgio and Cole, 2000). Although the bacterial production was estimated from the increase in bacterial abundance during exponential growth in the jellyfish treatments, the bacterial production estimate is well within the range reported in previous studies on microbial degradation of *A. aurita* detritus applying the standard ^3^H-leucine incorporation method (Tinta et al., 2010, 2012; Blanchet et al., 2015). The similar bacterial production estimates in this and previous studies also indicates that the use of freeze-dried material, as used in this study, compared to that of homogenized jellyfish carcasses (Tinta et al., 2010, 2012) or the <0.2 μm fraction of jelly-DOM (Blanchet et al., 2015) induced a similar response of the bacterial community. However, we do acknowledge that the use of freeze-dried material might affect the rate of the processing this jelly-OM by increasing the surface area and thus the accessibility of this material to marine microbes.

The high BGE (65 ± 27%) indicates that jelly-OM is efficiently incorporated into bacterial biomass, which is then accessible to bacterial grazers. This has important implications for the fate and flux of jellyfish-derived OM and for marine ecosystem functioning and its biogeochemical state. In contrast, the study of Condon et al. (2011) found that most DOM released by jellyfish is respired by bacteria rather than incorporated into bacterial biomass. However, as also stated in Condon et al. (2011), there is a major difference between DOM released by jellyfish while alive (*i.e.*, colloidal material with a C:N ratio of 25.6 ± 31.6:1, Condon et al., 2011; Dinasquet et al., 2013) and OM in jellyfish biomass and detritus (low C:N ratio and rich in proteins). In addition, the composition, stoichiometry and thus the bioavailability of jelly derived DOM might be species-specific (*i.e.*, jelly-DOM of *A. aurita* in our study vs. *Chrysaora quinquecirrha* and ctenophores *Mnemiopsis leidyi* studied by Condon et al., 2011; Dinasquet et al., 2013). Yet, our findings contrast those of Blanchet et al. (2015) studying the response of the bacterial community from a coastal lagoon to the DOM fraction of *A. aurita* reporting a BGE <20%. The overall environmental conditions might affect the microbial response to jellyfish OM, as our study was performed with water collected from a coastal oligotrophic system (northern Adriatic), while the study of Blanchet et al. (2015) was conducted in a eutrophic lagoon. Also, Blanchet et al. (2015) used jellyfish DOM (<0.2 μm fraction) of juvenile medusae kept in captivity.

### Bacterial Processing of Jellyfish Detritus Has Implications for the Biogeochemical Cycles

In our experiments, the C:N ratio of the DOM pool increased from 3.4 to 5.5 in the jelly-OM treatment within 1.5 d (Figures 1B,D and Supplementary Figure S7) and only ∼2% of all soluble jelly proteins were left in the media (Figure 3 and Supplementary Table S5). At the same time, bacteria consumed >70% of jelly-AA pool with net uptake rate of 186 ± 9 nmol L^–1^ h^–1^. Our results show that bacteria preferred the more easily accessible DFAA, since they consumed ∼97% of the originally available jelly-DFAA pool within first 2 d. Simultaneously, the accumulation of some DFAA species was detected (i.e., leucine reaching a concentration of 3.9 ± 0.2 μmol L^–1^ after 1.5 d, Supplementary Figure S2), probably resulting from bacterial cleavage of jellyfish proteins. The substantial release of DFAA, as a result of bacterial processing of jellyfish detritus, can have important implications for the functioning and biogeochemical state of the ecosystem. This is particularly true for coastal ecosystems, such as the northern Adriatic, where we showed that ambient AA pool is mostly (>88%) composed of DCAA (Figure 1C and Supplementary Figure S2B).

When bacteria entered the decay phase, only ∼0.1% of the jellyfish proteins from the originally present proteins were detected, indicating a rapid turnover rate of most soluble jellyfish proteins (Figure 3 and Supplementary Table S5). Some soluble jellyfish proteins (and likewise some jelly-DON compounds), in particular, actin- and titin-like proteins, are apparently more resistant to bacterial degradation (Figure 3 and Supplementary Table S5). At the same time, by-products of bacterial processing of proteinaceous jelly-OM were accumulating in the media as indicated by the increase in DCAA and tryptophan in the jelly-OM treatment (Figure 1 and Supplementary Figure S2). Tryptophan has been recognized as a major metabolite in particles down to 150 m depths (Johnson et al., 2020). Microbial utilization of jelly-OM resulted in an increase in NH_4_^+^ and PO_4_^3–^ in the batch cultures (Figures 1E,F), as previously reported (Tinta et al., 2010, 2012; Blanchet et al., 2015). Thus, the decay of jellyfish blooms in coastal waters might rapidly increase the concentrations of major inorganic nutrients, which, in turn, might lead to nuisance phytoplankton blooms in coastal waters. As jellyfish bloom decay occurs mainly in late spring and summer in temperate coastal waters when the water column is stratified and low turbulence conditions prevail in surface waters, ideal conditions are provided for bacterial utilization of jelly-OM to induce harmful phytoplankton blooms.

## Conclusion

We found that about half of the jelly-OM pool consists of labile DOM, essentially exclusively accessible to marine microorganisms. The jelly-DOM pool is consumed within ∼1.5 d by a consortium of opportunistic bacteria, including the genera *Pseudoalteromonas*, *Alteromonas*, and *Vibrio*. Interestingly, these bacteria are frequently associated with living jellyfish, in particularly with the mucus covering the jellyfish body (Tinta et al., 2019). This jellyfish degrading bacterial consortium exhibits a high growth efficiency. This has important implications for the fate of jelly-OM, which is apparently efficiently retained in the pelagic food web. We estimate that half of the jelly-OM pool is degraded and incorporated into planktonic bacterial biomass and remineralized in the water column. This implies that the amount of jelly-OM reaching the seafloor is effectively reduced by microbial processing of jelly-OM in the water column.

## Data Availability Statement

All data needed to evaluate the conclusions of the article are present in the article and/or the Supplementary Material. Raw reads of all metagenomic DNA libraries were deposited at NCBI under the accession number PRJNA633735. The proteomic raw data was were deposited at ProteomeXchange under accession number PXD021342 and at jPOST under accession number JPST000960. Additional data related to this article may be requested from the corresponding author.

## Author Contributions

TT designed and conducted experiments, acquired data, performed data analysis, drafted, and submitted the final version of the manuscript. ZZ performed analysis of metagenomic and proteomic data. KK performed chemical analysis. AE conducted experiments and performed microscopy-based analysis. BB contributed and assisted with preparation of samples for metagenome sequencing. CA contributed and assisted with preparation of samples for microscopy-based analysis. LB performed UPLC-analysis. GJH designed experiments, drafted, and revised several versions of the manuscript. All authors contributed to the article and approved the submitted version.

## Conflict of Interest

The authors declare that the research was conducted in the absence of any commercial or financial relationships that could be construed as a potential conflict of interest.

## Footnotes

1 http://www.technobiology.ch/index.php?id=acmetool

2 www.viennabiocenter.org/facilities

3 www.r-project.org/
