# Supplementary material for: Microbial Processing of Jellyfish Detritus in the Ocean
Source: Front Microbiol. 2020 Oct 30;11:590995. doi: 10.3389/fmicb.2020.590995 (PMC7662693; doi:10.3389/fmicb.2020.590995)

Supplementary Material

Table S1: Dry weight (DW) of collected jellyfish specimens on specific sampling dates (dd/mo/yr).

Date	DW (g)
28/03/18	13.3
28/03/18	12.7
28/03/18	7.1
04/04/18	6.6
04/04/18	13.2
04/04/18	2.2
04/04/18	23.2
04/04/18	15.1
04/04/18	11.5
04/04/18	14.6
04/04/19	17.0
07/05/18	4.8
07/05/18	14.4
07/05/18	7.7
07/05/18	7.8
07/05/18	11.2
07/05/18	8.9
07/05/18	6.6
07/05/18	8.4
09/05/18	12.9
09/05/18	10.0
09/05/18	12.0
09/05/18	6.2
09/05/18	13.6
09/05/18	12.0
24/05/18	11.7
24/05/18	11.6

S Info 1: Dialysis of jelly-DM

Dialysis of jelly-DM was performed using Spectra/Por 7 Membrane tubing (Sulphur and Heavy Metal Free Spectrum) with a MWCO of 1000 Da using five replicate samples. Dialysis membranes were pre-processed, prepared and used according to the manufacturer's instructions (Spectrum) and sealed with Spectrum™ Universal Dialysis Tubing Closures (Spectrum). For each replicate 1 g of jelly-DM was re-hydrated in 25 mL of Milli-Q water to mimic the average wet weight of the collected specimen and poured into dialysis bag. The dialysis bags were submerged in an acid-cleaned and combusted glass beaker filled with Milli-Q water and stirred at low speed on magnetic stirrer. Dialysis of all five replicates was performed at 4°C in the dark for 24 h by changing Milli-Q water after 2, 4, 6, 10 and 24 h until salinity reached 0. Salinity was measured using a refractometer. At each time point the dialysate was subsampled for determining the concentrations of dissolved organic carbon (DOC), total dissolved nitrogen (TDN) and dissolved inorganic nitrogen (DIN = NH_4^+ + NO_3^- + NO_2^-). In addition, the amount of DOC and TDN leaching from a dialysis membrane submerged in Milli-Q water was also measured and the values of all parameters measured were subtracted from the values of parameters measured in the dialysate.

Table S2: The cumulative amount of DOC, TDN, DON, DIN and NH_4^+, NO_3^-. NO_2^- and PO_4^{3-} that leached through a 1000 Da Molecular Weight Cut-Off (MWCO) membrane tubing within 24 h expressed in nmol per mg of jelly-DM d^{-1}. Values are average values of five replicates (±SD). The fraction <1000 Da represents low molecular weight compounds (LMW). The amount of high molecular weight compounds (HMW, >1000 Da) is expressed as the percentage of total jelly-DM per pool of compounds.

	LMW	Total jelly-DM	HMW
	nmol per mg of jelly-DM d^{-1}	nmol per mg of jelly-DM d^{-1}	% of Total jelly-DM
DOC	26 (±4)	440 (±3)	94.2(±0.9)
TDN	12 (±1)	130 (±1)	90.9(±0.8)
DON	11 (±1)	120 (±1)	91.2(±0.9)
DIN	1.3 (±0.07)		
NH_4^+	1.1 (±0.05)		
PO_4^{3-}	1.0 (±0.1)		

Figure S1: Scheme of the jellyfish degradation experiment with analyzed parameters at each of the sampling time points of the experiments.

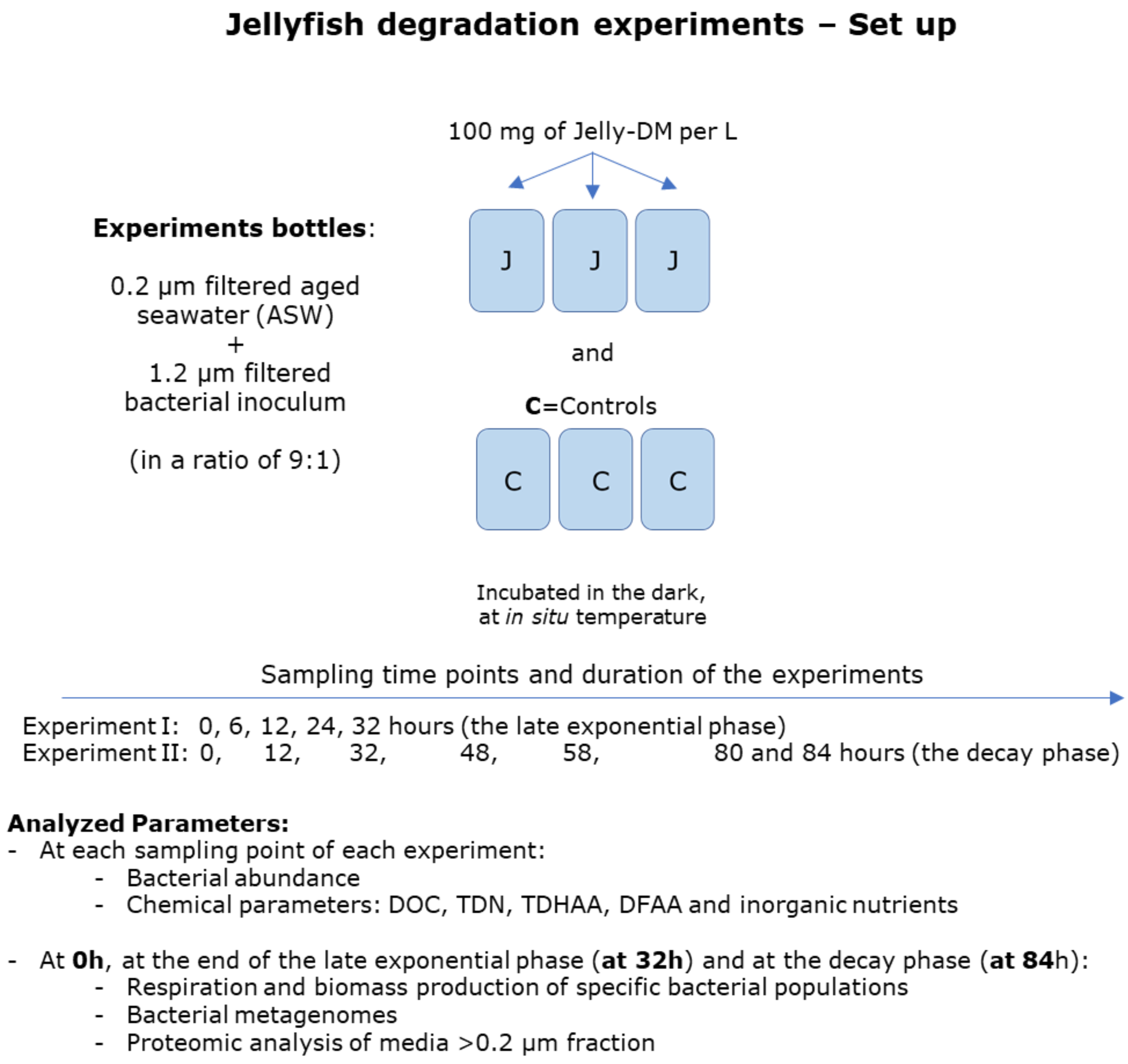

S info 2: Supplementary material to the protocol of Fluorescence *In Situ* Hybridization (FISH)

After embedding filters in 0.1% agarose at 37°C for 15 min, filters were washed with 96% ethanol, air-dried and incubated in 300 µL hybridization buffer (900 mM NaCl, 20 mM Tris-HCl, pH 8.0), 10% dextran sulfate, 0.05 % Triton, 1% blocking reagent, formamide (varying depending on the probe: 30% for G V, 35% for a mix of EUB338 I, II, III and 40% for alter2 and PSU730 in Sigma water) with 3 ng μL^{-1} of probe at 46°C for 3 h. After hybridization, filters were washed at 48°C in 50 mL pre-warmed washing buffer (20 mM Tris-HCl, pH 8.0, 5 mM EDTA, 0.01% SDS, NaCl (varied: 0.1 M for 30%, 0.08 M for 35% and 0.06 M for 40% formamide)) for 15 min. Afterwards, filters were washed with Milli-Q water, rinsed with 96% ethanol, air-dried and mounted with DAPI (see *Estimating bacterial abundance*).

Table S3: FISH oligonucleotide probes used in this study together with their sequence and the original publication.

**Probe**	**Sequence 5'-3'**	**Reference**
EUB338 I	GCT GCC TCC CGT AGG AGT	Amman *et al.* 1990
EUB338 II	GCA GCC ACC CGT AGG TGT	Daims *et al.* 1999
EUB338 III	GCT GCC ACC CGT AGG TGT	Daims *et al.* 1999
alter2	GGT CCG CTC CAC ATC ACT GT	Demanèche *et al*. 2008
PSU730	TTG ACC CAG GTG GCT GCC	Huggett *et al.* 2008
G V	AGG CCA CAA CCT CCA AGT AG	Eilers *et al.* 2000

References:

Amann, R. I., B. J. Binder, R. J. Olson, S. W. Chisholm, R. Devereux, and D. A. Stahl. 1990. Combination of 16S rRNA-targeted oligonucleotide probes with flow cytometry for analyzing mixed microbial populations. *Applied and environmental microbiology* 56: 1919–1925.

Daims, H., A. Brühl, R. Amann, K.-H. Schleifer, and M. Wagner. 1999. The Domain-specific Probe EUB338 is Insufficient for the Detection of all Bacteria: Development and Evaluation of a more Comprehensive Probe Set. *Systematic and Applied Microbiology* 22: 434–444. doi:10.1016/S0723-2020(99)80053-8

Demanèche, S., H. Sanguin, J. Poté, and others. 2008. Antibiotic-resistant soil bacteria in transgenic plant fields. *Proceedings of the National Academy of Sciences* 105: 3957. doi:10.1073/pnas.0800072105

Eilers, H., J. Pernthaler, F. O. Glöckner, and R. Amann. 2000. Culturability and In situ abundance of pelagic bacteria from the North Sea. *Applied and environmental microbiology 66*: 3044–3051. doi:10.1128/aem.66.7.3044-3051.2000

Huggett, M. J., G. R. Crocetti, S. Kjelleberg, and P. D. Steinberg. 2008. Recruitment of the sea urchin Heliocidaris erythrogramma and the distribution and abundance of inducing bacteria in the field. *Aquatic Microbial Ecology* 53: 161–171.

S Info 3: Supplementary material to dissolved amino acid analysis

Additional information on the protocol for amino acid analysis performed on a Shimadzu Nexera X2 ultra high-performance liquid chromatograph (UHPLC):

The concentration of dissolved combined amino acids (DCAA) was calculated as the difference between TDHAA and DFAA. Samples for TDHAA analysis were hydrolyzed as described by (Kaiser and Benner, 2005) with the following modifications: 382 µL of sample was pipetted into acid-, Milli-Q water rinsed and combusted glass ampules together with 608 µL of Suprapur 30% HCl (Merck) and 10 µL of 12 mM ascorbic acid, flushed with ultrapure nitrogen (grade 5), sealed with aluminum caps and incubated on a hot plate at 110°C for 20 h. Afterwards, samples were filtered through an 0.45 µm filter unit (PALL Inc) and dried with a stream of ultrapure nitrogen. Samples were brought back to 500 µL volume with Rotisolv Ultra LC MS Water (ROTH).

Pre-column derivatization was applied with ortho-phthalaldehyde (OPA) according to the protocol of Jones et al. (Jones et al., 1981), with slight modifications. Briefly, 2.5 µL of OPA (10 mg mL^{-1}, Agilent Technologies, USA) with 37 µL of borate buffer (0.4 N, pH 10.2, Agilent Technologies, USA) was added per 500 µL of sample or standard. Five µL of sample or standard was injected first through the pre-column (YMC, 5 x 3mm inner diameter, Triart ExRS, S- 1.9 µm, 8nm) and then fluorescent derivatives were separated on a YMC- Triart C18 ExRS column (Particle size - 1.9 µm, pore size - 8nm, 3 x 100 mm I.D.). The column temperature was set at 35°C and a flow rate of 0.5 mL min^{-1} was applied. The excitation and emission wavelengths were 340 nm and 450 nm, respectively. Mobile phases were 20 mM monobasic phosphate buffer (pH 6.5) and acetonitrile/methanol/water (45/40/15). Tetrahydrofuran was added to facilitate the separation of the individual compounds. The elution gradient program is provided below. With each batch of samples, amino acids standards AAS18 (2.5 µM stock, Sigma) were run in the range between 5 and 200 nM, distributed over the sequence run to account for a possible instrument drift. The limit of detection varied from 0.09 to 5.16 nM (see below). The procedural blanks were subtracted from the measured concentrations of amino acids. During hydrolysis, glutamine (Gln) and asparagine (Asn) react to glutamic acid (Glu) and aspartic acid (Asp), respectively, and are therefore reported as the sum (Asx and Glx). Methionine, tryptophan and isoleucine were inefficiently recovered after hydrolysis and were therefore omitted from further analysis. The recovery of other amino acid species after hydrolysis was about 100%.

Elution gradient program:

A: Monobasic phosphate 20mM, pH 6.5

B: Acetonitrile/Methanol/Water = 45/40/15

Time (min)	Solvent	Concentration (%)
2	B	11
	A	89
4	B	17
	A	73
5.5	B	31
	A	69
10	B	32.5
	A	67.5
12	B	46.5
	A	43.5
15.5	B	55
	A	43.5
15.51	B	100
	A	0
19.5	B	100
	A	0
20	B	100
	A	0
21	B	30
	A	70
22	B	11
	A	89
23	B	11
	A	89
25		STOP

The order of elution followed: 1- ASP, AC 2- GLU AC, 3- ASN, 4- SER, 5- GLN, 6-HIS, 7- GLY, 8- THR, 9- ARG, 10- ALA, 11- TAU, 12- GABA, 13- TYR, 14- VAL, 15- MET, 16- TRP, 17- PHE, 18-ILE, 19- LEU and 20- LYS

Limit of detection for individual amino acids (AA):

AA	nmol L^{-1}
LYS	5.16
TRP	2.18
MET	1.73
LEU	1.56
VAL	0.68
GLN	0.60
ASN	0.58
GABA	0.56
ARG	0.52
GLY	0.48
TYR	0.45
ALA	0.43
THR	0.43
TAU	0.33
ISO	0.33
PHE	0.29
SER	0.22
GLU AC	0.20
ASP AC	0.11
HIS	0.10

Table S4: The concentration of the individual dissolved free amino acid (DFAA) and dissolved combined amino acid (DCAA) fraction leaching from *A. aurita* dry matter given in nmol (mg jelly-DM) $^{-1}$ d^{-1} and as mol%. TDHAA stands for total dissolved hydrolyzable amino acids.

AA	DFAA nmol	DCAA nmol	DFAA mol%	DCAA mol%
**Asx***	0.36	4.62	0.60	11.14
**Glx****	2.91	12.57	4.87	30.30
**Asn**	0.45	n.d.	0.76	n.d.
**Ser**	0.58	2.83	0.97	6.81
**Gln**	1.14	n.d.	1.91	n.d.
**His**	0.29	0.67	0.49	1.61
**Gly**	25.11	10.22	41.98	24.64
**Thr**	1.18	n.d.	1.98	n.d.
**Arg**	0.46	2.55	0.77	6.14
**Ala**	0.85	6.47	1.42	15.59
**Tau**	22.61	0.00	37.80	0.00
**Tyr**	0.42	0.26	0.71	0.63
**Val**	0.69	n.d.	1.16	n.d.
**Met**	0.21	n.d.	0.36	n.d.
**Val+Met**	n.d.	4.98	n.d.	2.18
**Trp**	0.11	n.d.	0.19	n.d.
**Phe**	0.38	n.d.	0.64	n.d.
**Ile**	0.42	n.d.	0.71	n.d.
**Leu**	0.78	2.47	1.31	0.05
**Lys**	0.83	1.65	1.39	0.90
**ΣAA**	59.80	49.29	100	100
**TDHAA**	12.96		109.09	

* Asx, sum of aspartic acid and asparagine; ** Glx, sum of glutamic acid and glutamine; n.d., not detected.

Figure S2: (**A**) DFAA (top panels) and DCAA (bottom panels) dynamics (expressed as mol% (left panels) and in nmol L^{-1} (right panels)) in jelly-OM treatments during the course of both experiments and (**B**) the concentration of TDHAA (µmol L^{-1}) and the contribution of DFAA and DCAA to the TDHAA pool in control treatments during the course of both experiments. Each value represents the mean of three biological replicates (±SD) for the sampling time in both experiments. The mean (±SD) from both experiments is given. On x-axes: jelly-OM at 0h and sampling time points throughout the experiments (6, 12, 24, 32, 46, 58, 80 and 84 h).

A)

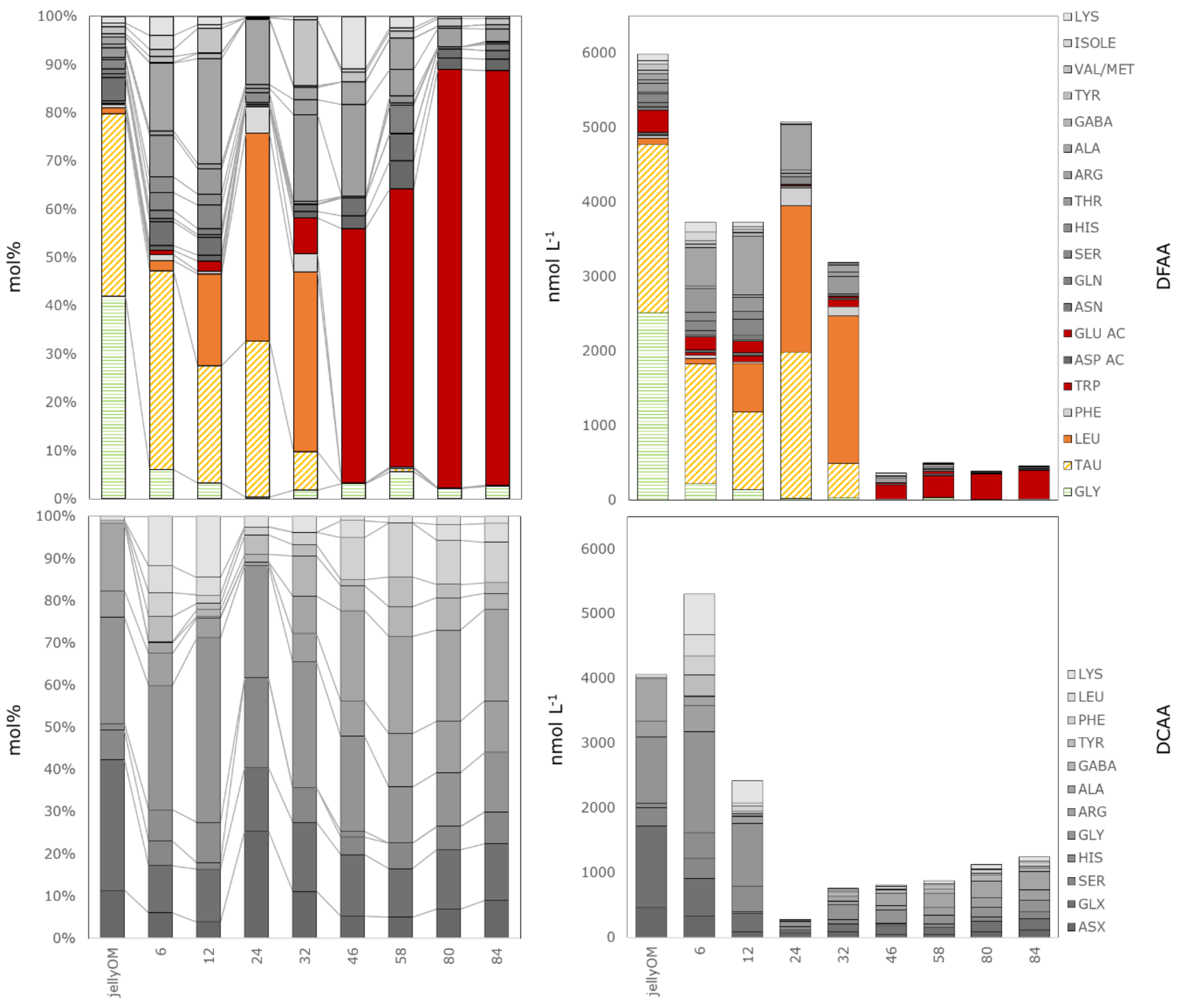

B)

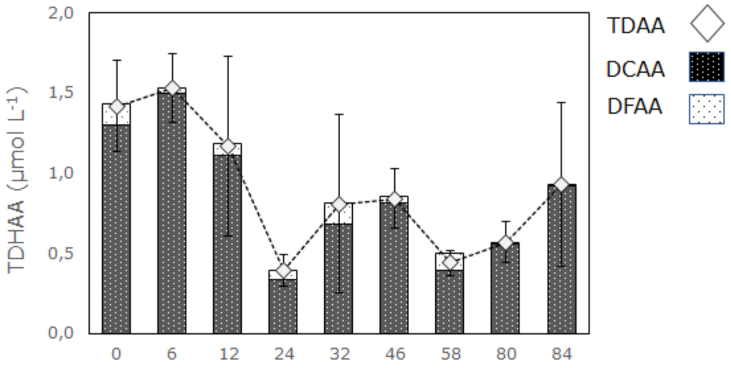

S Info 4: Supplementary material to proteomics analysis

Figure S3: COG functional categories of 2049 annotated out of 10966 jelly-DM proteins detected using Q Exactive™ Hybrid Quadrupole-Orbitrap™ Mass Spectrometer.

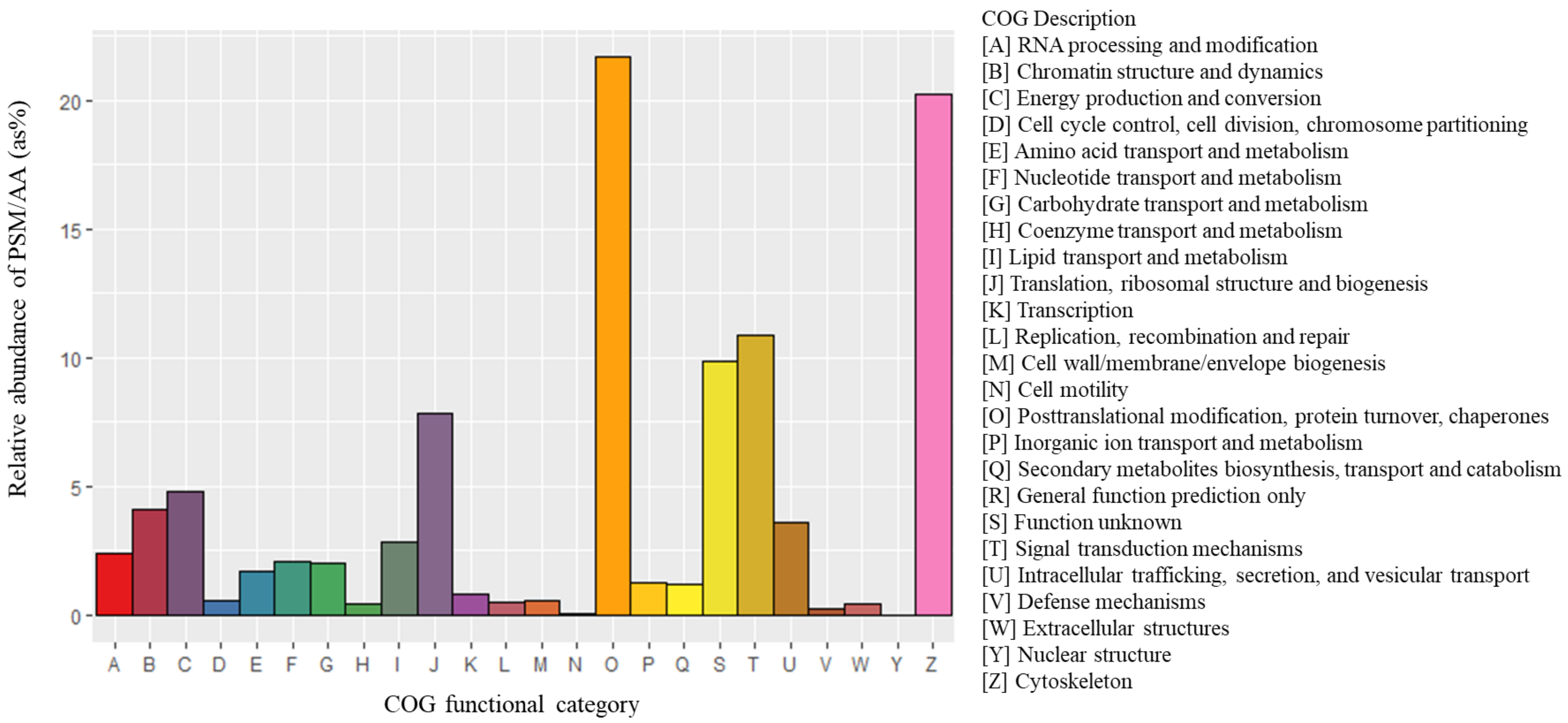

Table S5: S5A) List of all soluble proteins extracted and sequenced from 100 mg of jelly-DM and their corresponding PSM/AA values, **S5B**) the list of 2820 unique proteins assigned to 6978 annotated proteins, their corresponding PSM/AA values and the relative abundance, and **S5C**) the jelly-proteins detected and annotated in the 0.2 µm – 5000Da fraction of the media collected from jellyfish treatments at the peak of bacterial abundance (at 32 h, Experiment I) and at the decay phase of bacterial growth (at 84 h, Experiment II) with their PSM/AA value at each time point and their relative abundance within the corresponding jelly-protein pool. The table is provided as a separate file.

Supplementary to LC-MS/MS analysis and peptide identification protocol

LC-MS/MS analysis and peptide identification were performed as previously described in detail (Bayer et al., 2019a) with slight modifications. In brief: 5 µL of desalted peptides were eluted using an Easy-spray PepMap RSLC column (ThermoFisher Scientific, C18, 500 mm x 75 µm, pore size 2.0 µm) during a 270-min gradient from 5 to 40% (v/v) acetonitrile and 0.1% (v/v) formic acid with a controlled flow rate of 300 nL min^{-1}. MS analysis was performed on a Q-Exactive™ Hybrid Quadrupole-Orbitrap™ Mass Spectrometer (Thermo Fisher Scientific) with specific settings as described by (Bayer et al., 2019a).

S Info 5: Supplementary material to the bacterial metagenome analysis

Protocol for extraction of total nucleic acids

Total nucleic acids were extracted from the filters following the protocol of Angel (Angel, 2012) with some modifications. Filters were ground into small pieces with a sterile metal spatula after submerging the tubes with the filters into liquid nitrogen. Phosphate buffer (750 µL, 120 mM, pH 8) and 250 µL of TNS buffer (500 mM Tris base, 100 mM NaCl, 10% SDS) were added to filter pieces and cells were lysed with five freeze-and-thaw cycles. After centrifugation (20,000 *g* at 4°C for 3 min) the supernatant was transferred into a tube and 1 volume of TE-saturated phenol (pH 8) was added. DNA was purified using the standard phenol/chloroform/isoamyl alcohol and chloroform/isoamyl alcohol protocol. Due to the high pH used in our protocol (pH 8), most of our nucleic acids was dsDNA, especially, since we did not take any precautions for RNA preservation. Nucleic acids were precipitated with 30% polyethylene glycol in 1.6 M NaCl and 2 µL glycogen (Roche), washed once with ice-cold 75% EtOH and resuspended in low TE buffer, pH 8. The concentration of dsDNA was estimated with Quant-iT™ PicoGreen™ dsDNA Assay Kit (ThermoFischer).

Overview of metagenomic assembly including number of reads, number of contigs (after MEGAHIT), the average shortest contig length needed to cover 50% of the genome (i.e. N50(bp)) and number of predicted genes (after Prodigal).

Sample	No. of reads	No. of contig	N50(bp)	No. of predicted genes
Coastal	64147456	713288	1447	1257630
Control	65597288	561450	1314	927297
Jelly-OM	62203173	177993	2411	324588

Table S6: Diversity indices of the bacterial community classified at the class and family level based on metagenome taxonomic profiling of the coastal microbial community and communities in the jellyfish and control treatments at the peak of bacterial abundance.

Diversity at class level	Coastal	Control treatments	Jelly-OM treatments
S (Species richness)	154	127	62
H (Shannon–Wiener)	1.15	0.93	0.36
J (Pielou's evenness)	0.23	0.19	0.07
Simpson (1-D)	0.55	0.50	0.19

Diversity at family level	Coastal	Control treatments	Jelly-OM treatments
S (Species richness)	558	448	227
H (Shannon–Wiener)	2.18	1.96	1.41
J (Pielou's evenness)	0.43	0.39	0.28
Simpson (1-D)	0.76	0.76	0.68

Figure S4: Taxonomic profiling of the metagenome of the coastal microbial community and communities in the jellyfish and control treatments at the peak of bacterial abundance. **A** – relative abundance (%) of bacteria classified at the class level. **B** – relative abundance (%) of bacteria classified at the family level.

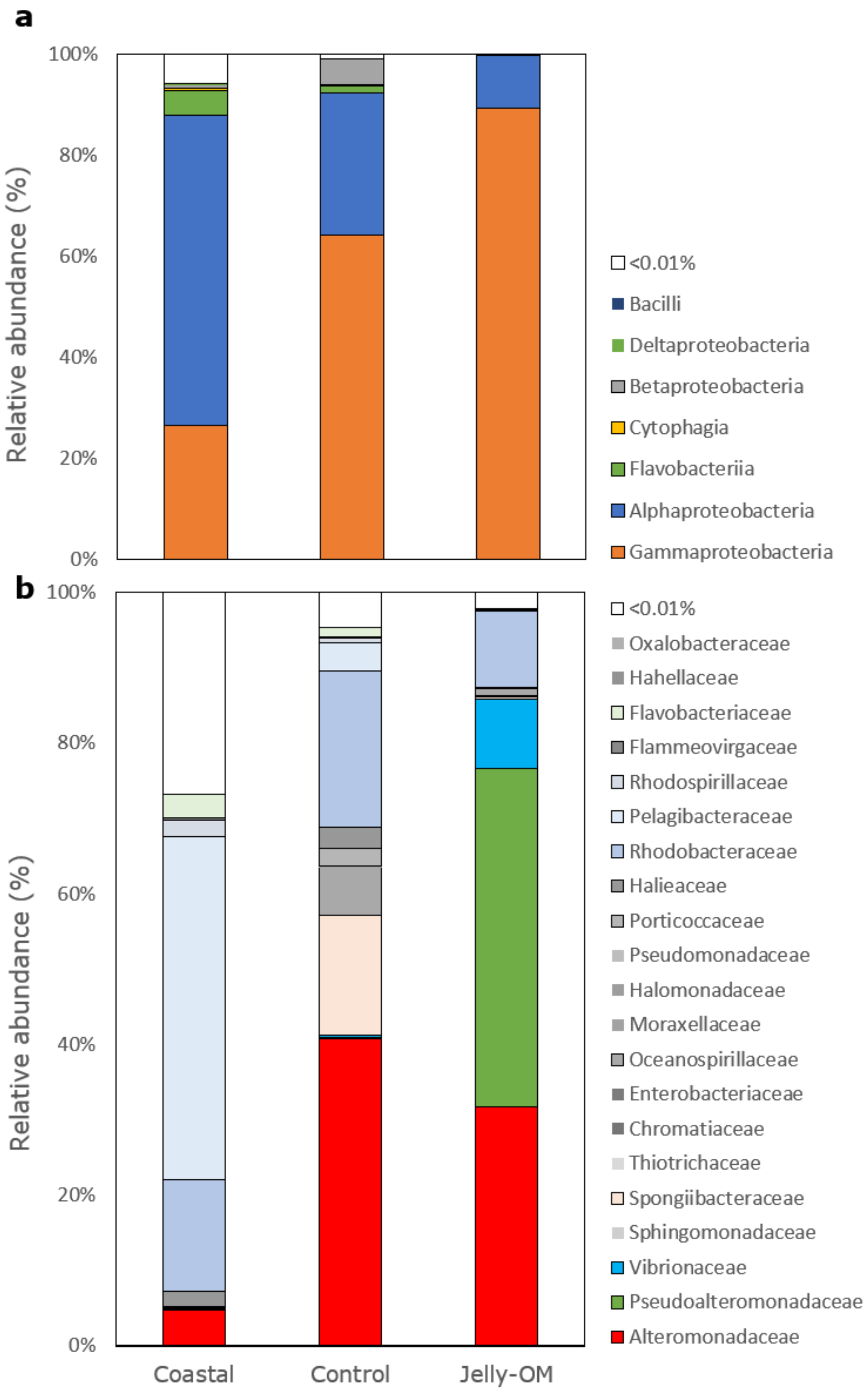

Figure S5: **A)** Relative abundance of reads per million mapped reads (RPMs) of 84 MAGs assembled from the coastal microbiome, control and jelly-OM treatment metagenomes at the at the peak of bacterial abundance. The abundance of MAGs identified as major jelly-OM degraders, affiliating to *Pseudoalteromonas*, *Alteromonas* and *Vibrio* is highlighted in green, red and blue, respectively, all the remaining are in grey scale. RPM-based relative abundance is the percentage based on the RPM of a specific MAG divided by the sum of the RPM of all MAGs.

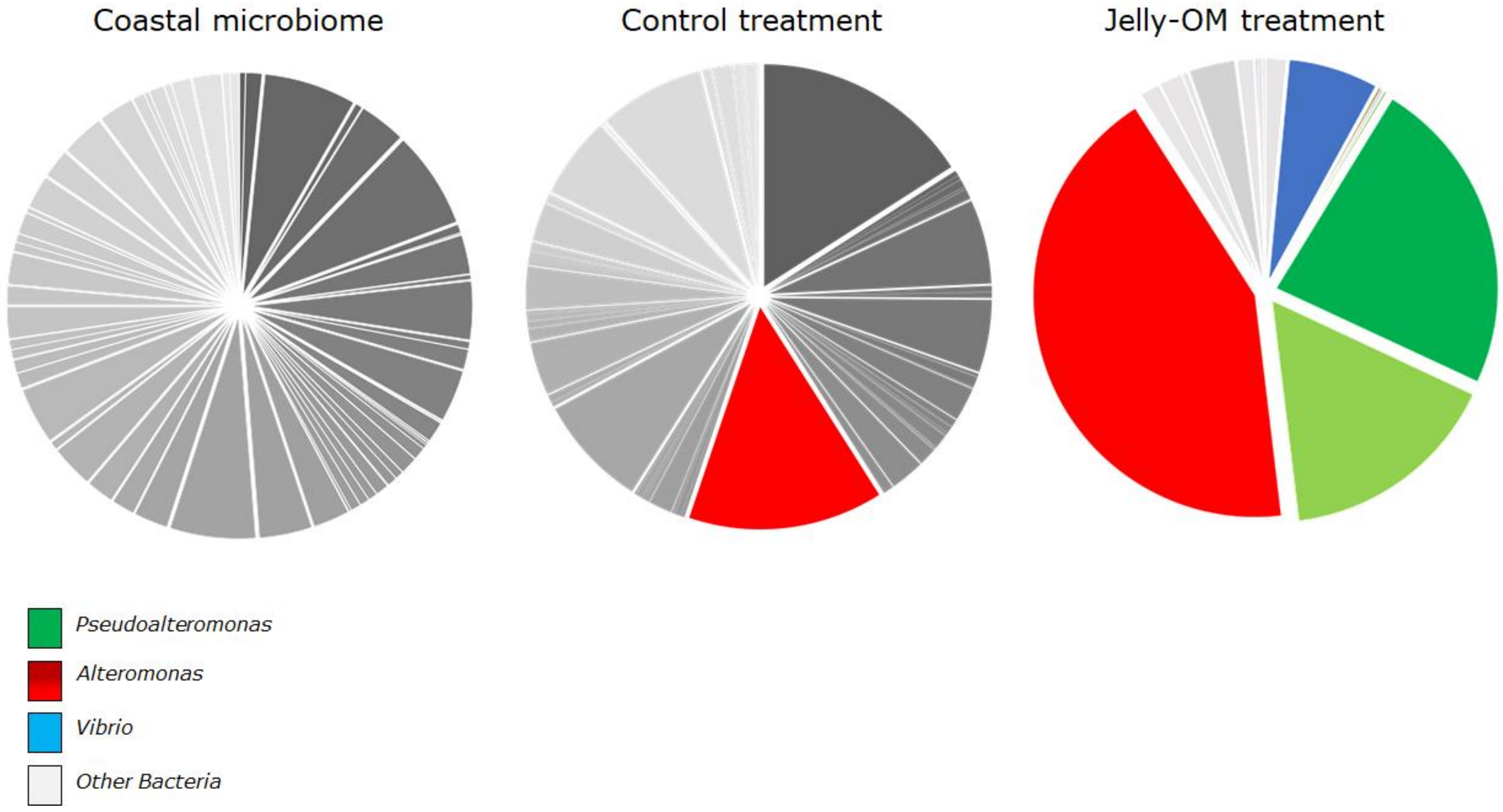

B) Reads per million mapped reads (RPM) of all 84 MAGs assembled from the coastal microbiome, control and jelly-OM treatment metagenomes at the peak of bacterial abundance and classified at the family level. The MAGs RPMs of families of the major jelly-OM degraders, *Pseudoalteromonadaceae*, *Alteromonadaceae* and *Vibrionaceae*, all belonging to Gammaproteobacteria, are highlighted in green, red and blue, respectively, while all the remaining bacterial families are summed as Others in grey scale.

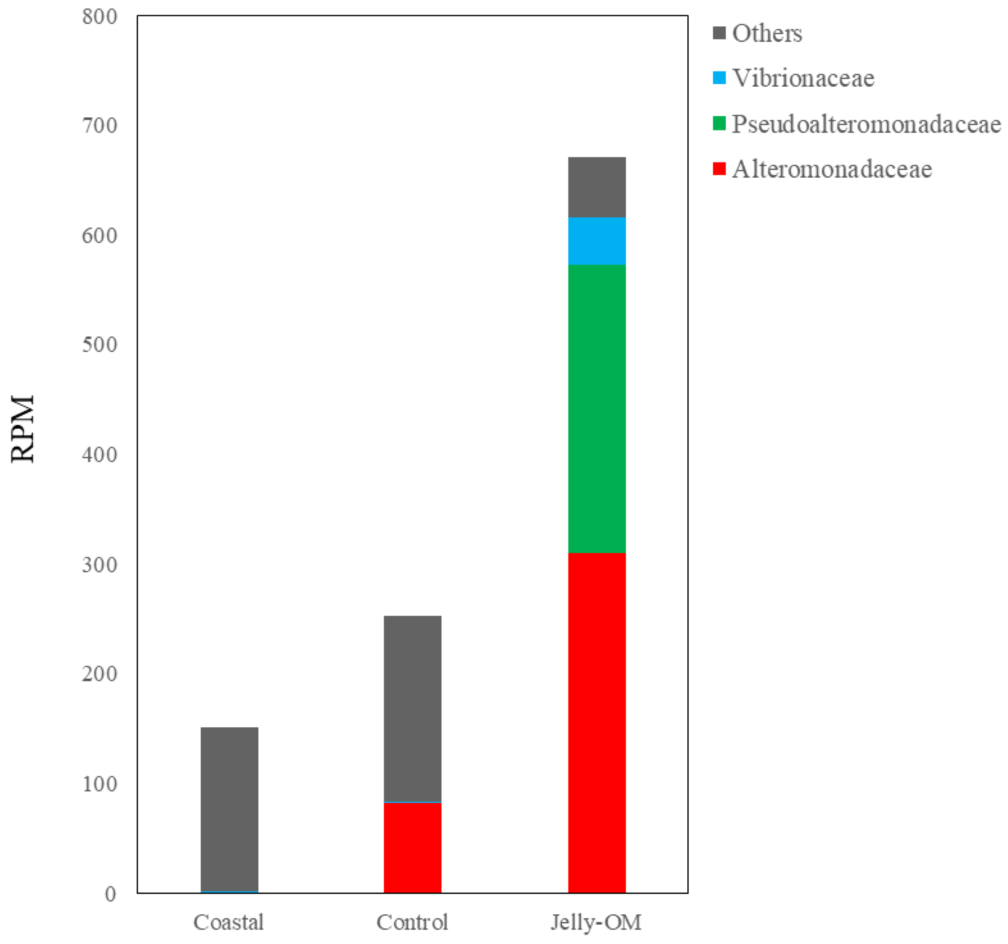

Table S7: Microscopy-based analysis of specific bacterial populations. A - DAPI counts (*i.e.*, total cell abundance), percentage of bacteria (cells hybridized with the EUBI, II, III FISH probe) and the percentage of specific bacterial populations in the coastal assemblage (average of two biological replicates ±SE), at the peak of the abundance (*i.e.*, late exponential phase, Experiment I) and in the decay phase (Experiment II) in the control and jelly-OM treatment (each value represents the mean of three biological replicates ±SD) as determined by specific FISH probes (Table S2). **B** - Comparison of the abundance of specific bacterial populations determined by FISH in the control *vs* jelly-OM treatment in the late exponential and decay phase of bacterial community growth (p-values, Student's t-test).

**A**		Late exponential phase		Decay phase	
	Coastal	Control	Jelly-OM	Control	Jelly-OM
Total cells (x10^5 cells mL^{-1})	5.7 ± 1.1	9.9 ± 4.3	114 ± 3.2	4.5 ± 1.9	12.4 ± 3.8
% of Bacteria	32 ± 9 *	67 ± 31	98 ± 18	62 ± 25	71 ± 7
% *Pseudoalteromonas*	9.5 ± 4.2	16 ± 18	50 ± 16	4 ± 3	16 ± 15
% *Alteromonas*	8.4 ± 0.7	14 ± 7	31 ± 10	10 ± 8	28 ± 28
% *Vibrio*	3.1 ± 1.7	4 ± 4	11 ± 2	6 ± 6	13 ± 12

**B**	Late exponential phase	Decay phase
Bacteria	0.0001	0.0029
Pseudoalteromonas	0.0045	0.0007
Alteromonas	0.0005	0.1288
Vibrio	0.0007	0.0288

***Note:** The low percentage of bacteria in the coastal assemblage is most likely due to the use of the standard fluorescence *in situ* hybridization method rather than the CARD-FISH method. Combining the CARD-FISH protocol with click-iT chemistry or the Redox Sensor Green reagent was not possible.

Table S8: **Microscopy-based analysis of respiring and HPG incoporating bacterial populations. A -** Abundance of total active bacterial cells (*i.e.*, cell was considered actively respiring/HPG incorporating if overlaying DAPI and RSG/HPG signal was present) and the percentage of active bacterial populations (*i.e.*, bacterial cell with overlaying signal for DAPI, specific FISH probe and RSG or HPG) in the coastal assemblage (average of two biological replicates ±SE) at the peak of the abundance (*i.e.*, late exponential phase, Experiment I) and in the decay phase (Experiment II) in the control and jelly-OM treatment (mean of three biological replicates ±SD) as determined using specific FISH probes (Table S2). **B** - Comparison of the abundance of metabolically active bacterial populations in the control *vs* jelly-OM treatment in the late exponential and decay phase of bacterial community growth (p-values, Student's t-test).

**A**		**Respiring community**			
		Late exponential phase		Decay phase	
	Coastal	Control	Jelly-OM	Control	Jelly-OM
Total active cells (x10^5 cells mL^{-1})	2.5 ± 0.6	4 ± 0.7	48 ± 5	3 ± 0.6	4 ± 0.8
% of active Bacteria	54 ± 14	81 ± 15	98 ± 12	69 ± 18	88 ± 21
Active Bacteria (x10^5 cells mL^{-1})	1.4 ± 0.6	3 ± 0.6	47 ± 6	2 ± 0.5	4 ± 0.9
% of active *Pseudoalteromonas*	10 ± 4	2 ± 0.3	50 ± 6	1 ± 0.4	4 ± 1
% of active *Alteromonas*	13 ± 3	13 ± 3	37 ± 4	4 ± 1	47 ± 12
% of active *Vibrio*	4 ± 2	1 ± 0.1	12 ± 1	1 ± 0.4	6 ± 2

**A**		**HPG incorporating community**			
		Late exponential phase		Decay phase	
	Coastal	Control	Jelly-OM	Control	Jelly-OM
Total active cells (x10^5 cells mL^{-1})	2.2 ± 1.5	4 ± 3	42 ± 12	1.6 ± 0.9	5 ± 0.2
% of active Bacteria	48 ± 32	91 ± 52	99 ± 29	45 ± 27	93 ± 0.3
Active Bacteria (x10^5 cells mL^{-1})	1.3 ± 1.4	4 ± 2	41 ± 12	0.5 ± 0.3	4 ± 0.02
% of active *Pseudoalteromonas*	3 ± 1	32 ± 37	53 ± 14	11 ± 8	41 ± 0.2
% of active *Alteromonas*	5 ± 4	19 ± 21	28 ± 7	20 ± 14	29 ± 0.1
% of active *Vibrio*	13 ± 15	7 ± 8	11 ± 3	17 ± 12	29 ± 0.1

**B**	Respiration		HPG incorporation	
	Late exponential phase	Decay phase	Late exponential phase	Decay phase
Bacteria	0.0002	0.0293	0.0061	0.0061
Pseudoalteromonas	0.0029	0.0879	0.0181	0.0181
Alteromonas	0.0001	0.2620	0.0200	0.0200
Vibrio	0.0026	0.0919	0.0002	0.0002

Table S9: DOC concentration (μmol L^{-1}) in the jelly-OM (J1, J2, J3) and control (C1, C2, C3) treatments in Experiment I and Experiment II during the course of incubation. n.a.– not analyzed

	Experiment I						Experiment II					
T (h)	C1	C2	C3	J1	J2	J3	C1	C2	C3	J1	J2	J3
0	87.9	n.a.	n.a.	n.a.	n.a.	n.a.	87.9	82.1	92.3	135.3	123.3	118.8
6	74.8	82.2	80.3	110.7	114.3	114.7	n.a.	n.a.	n.a.	n.a.	n.a.	n.a.
12	76.2	80.6	79.6	181.2	120.9	120.9	79.3	79.1	80.1	110.8	98.2	100.7
24	80.9	81.9	80.2	136.2	119.3	100.0	n.a.	n.a.	n.a.	n.a.	n.a.	n.a.
32	82.8	87.9	80.9	89.5	108.4	90.3	84.4	85.6	76.3	96.3	107.8	96.5
46	n.a.	n.a.	n.a.	n.a.	n.a.	n.a.	91.3	91.3	103.6	98.5	96.3	104.9
58	n.a.	n.a.	n.a.	n.a.	n.a.	n.a.	81.0	79.3	129.1	95.7	94.7	102.1
80	n.a.	n.a.	n.a.	n.a.	n.a.	n.a.	84.6	78.2	79.4	104.9	105.5	108.4
84	n.a.	n.a.	n.a.	n.a.	n.a.	n.a.	96.1	89.4	80.8	96.5	116.3	297.2

Figure S6: **A**) Dynamics of virus-like particles and **B**) microscopy image of respiring protists in the treatments at the end of the experiment indicated by red arrows.

A)

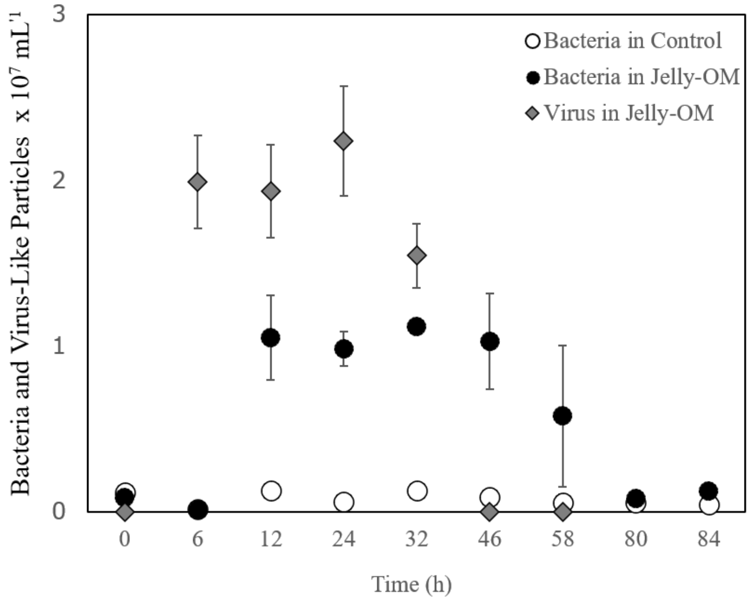

B)

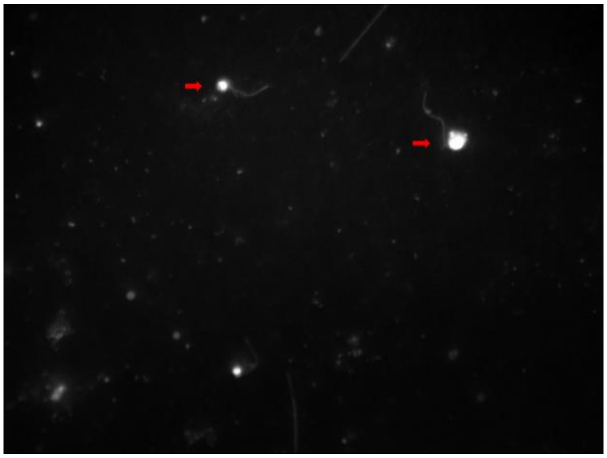

The enumeration of protists in the experiment was not performed. However, based on the observation, the number of protists increased towards the end of the experiments.

Figure S7: Dynamics of the C : N ratio throughout the experiment in the jelly-OM and control treatment.

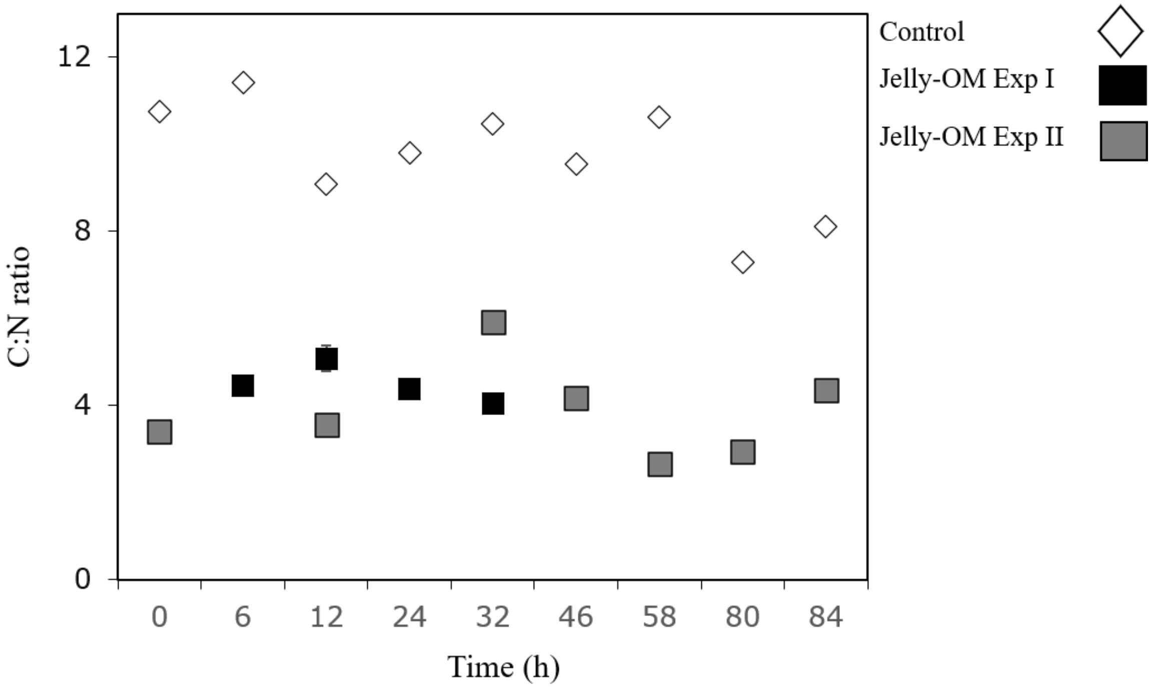

Supplement: Supplementary file 1 [file Data_Sheet_1.zip › Supplementary Material/Supplementary Material_Main.pdf]
